# ﻿Phylogenomic analysis of 997 nuclear genes reveals the need for extensive generic re-delimitation in Caesalpinioideae (Leguminosae)

**DOI:** 10.3897/phytokeys.205.85866

**Published:** 2022-08-22

**Authors:** Jens J. Ringelberg, Erik J. M. Koenen, João R. Iganci, Luciano P. de Queiroz, Daniel J. Murphy, Myriam Gaudeul, Anne Bruneau, Melissa Luckow, Gwilym P. Lewis, Colin E. Hughes

**Affiliations:** 1 Department of Systematic and Evolutionary Botany, University of Zurich, Zollikerstrasse 107, CH 8008, Zurich, Switzerland University of Zurich Zurich Switzerland; 2 Present address: Evolutionary Biology & Ecology, Université Libre de Bruxelles, Faculté des Sciences, Campus du Solbosch - CP 160/12, Avenue F.D. Roosevelt, 50, 1050 Bruxelles, Belgium Université Libre de Bruxelles Bruxelles Belgium; 3 Instituto de Biologia, Universidade Federal de Pelotas, Campus Universitário Capão do Leão, Travessa André Dreyfus s/n, Capão do Leão 96010-900, Rio Grande do Sul, Brazil Universidade Federal de Pelotas Rio Grande do Sul Brazil; 4 Programa de Pós-Graduação em Botânica, Universidade Federal do Rio Grande do Sul, Avenida Bento Gonçalves, 9500, Porto Alegre, Rio Grande do Sul, 91501-970, Brazil Universidade Federal do Rio Grande do Sul Rio Grande do Sul Brazil; 5 Departamento Ciências Biológicas, Universidade Estadual de Feira de Santana, Avenida Transnordestina s/n – Novo Horizonte, 44036-900, Feira de Santana, Brazil Universidade Estadual de Feira de Santana Feira de Santana Brazil; 6 Royal Botanic Gardens Victoria, Birdwood Ave., Melbourne, VIC 3004, Australia Royal Botanic Gardens Victoria Melbourne Australia; 7 Institut de Systématique, Evolution, Biodiversité (ISYEB), MNHN-CNRS-SU-EPHE-UA, 57 rue Cuvier, CP 39, 75231 Paris, Cedex 05, France Institut de Systématique, Evolution, Biodiversité (ISYEB) Paris France; 8 Institut de Recherche en Biologie Végétale and Département de Sciences Biologiques, Université de Montréal, 4101 Sherbrooke St E, Montreal, QC H1X 2B2, Canada Université de Montréal Montreal Canada; 9 School of Integrative Plant Science, Plant Biology Section, Cornell University, 215 Garden Avenue, Roberts Hall 260, Ithaca, NY 14853, USA Cornell University Ithaca United States of America; 10 Accelerated Taxonomy Department, Royal Botanic Gardens, Kew, Richmond, Surrey, TW9 3AE, UK Accelerated Taxonomy Department, Royal Botanic Gardens Richmond United Kingdom

**Keywords:** Fabaceae, generic delimitation, mimosoid clade, monophyly, morphological homoplasy, phylogenomics

## Abstract

Subfamily Caesalpinioideae with ca. 4,600 species in 152 genera is the second-largest subfamily of legumes (Leguminosae) and forms an ecologically and economically important group of trees, shrubs and lianas with a pantropical distribution. Despite major advances in the last few decades towards aligning genera with clades across Caesalpinioideae, generic delimitation remains in a state of considerable flux, especially across the mimosoid clade. We test the monophyly of genera across Caesalpinioideae via phylogenomic analysis of 997 nuclear genes sequenced via targeted enrichment (Hybseq) for 420 species and 147 of the 152 genera currently recognised in the subfamily. We show that 22 genera are non-monophyletic or nested in other genera and that non-monophyly is concentrated in the mimosoid clade where ca. 25% of the 90 genera are found to be non-monophyletic. We suggest two main reasons for this pervasive generic non-monophyly: (i) extensive morphological homoplasy that we document here for a handful of important traits and, particularly, the repeated evolution of distinctive fruit types that were historically emphasised in delimiting genera and (ii) this is an artefact of the lack of pantropical taxonomic syntheses and sampling in previous phylogenies and the consequent failure to identify clades that span the Old World and New World or conversely amphi-Atlantic genera that are non-monophyletic, both of which are critical for delimiting genera across this large pantropical clade. Finally, we discuss taxon delimitation in the phylogenomic era and especially how assessing patterns of gene tree conflict can provide additional insights into generic delimitation. This new phylogenomic framework provides the foundations for a series of papers reclassifying genera that are presented here in *Advances in Legume Systematics* (ALS) 14 Part 1, for establishing a new higher-level phylogenetic tribal and clade-based classification of Caesalpinioideae that is the focus of ALS14 Part 2 and for downstream analyses of evolutionary diversification and biogeography of this important group of legumes which are presented elsewhere.

## ﻿Introduction

In 2017, the Legume Phylogeny Working Group established a new subfamily classification of the Leguminosae (LPWG 2017), which dealt with the longstanding problem of the paraphyly of old sense subfamily Caesalpinioideae DC. by formally dividing the family into six subfamilies: Cercidoideae LPWG, Detarioideae Burmeist., Duparquetioideae LPWG, Dialioideae LPWG, Caesalpinioideae and Papilionoideae DC. Subfamily Caesalpinioideae was especially impacted by this new classification because several large clades previously included within it were afforded subfamily rank, while at the same time the former subfamily Mimosoideae DC., which is nested within Caesalpinioideae, was subsumed within the re-circumscribed Caesalpinioideae and is now simply referred to as the mimosoid clade (LPWG 2017). The idea that Leguminosae comprises six main lineages has since been amply confirmed by phylogenomic analyses of large nuclear gene and plastome DNA sequence datasets (Koenen et al. 2020a; Zhang et al. 2020; Zhao et al. 2021) providing robust support for the six subfamilies. Establishment of this new classification has shifted the focus of current legume systematics research to development of phylogenetically-based tribal (e.g. de la Estrella et al. 2018 for Detarioideae) and clade-based (e.g. Sinou et al. 2020 for Cercidoideae) higher-level classifications and, especially, towards establishment of robust generic systems for each subfamily. Here, we present a phylogenomic backbone for the re-circumscribed subfamily Caesalpinioideae as the basis for a new higher-level and generic classification of that subfamily.

Caesalpinioideae sensu LPWG (2017) is the second largest subfamily of legumes with ca. 4,600 species currently placed in 152 genera (LPWG 2017 plus additions, see below). Within this subfamily, ca. 3,400 species and 90 genera are placed in the mimosoid clade corresponding to the former subfamily Mimosoideae, which is nested within new sense Caesalpinioideae (LPWG 2017). Caesalpinioideae has a pantropical distribution and many of its lineages form ecologically abundant or dominant elements across each of the major lowland tropical biomes – seasonally dry tropical forests (“the succulent biome” sensu Schrire et al. 2005 and Ringelberg et al. 2020), savannas and tropical rain forests – thus spanning the full lowland tropical rainfall spectrum from arid to hyper-wet, with just a small fraction of species extending into the warm temperate zone, a subset of which are frost tolerant. Caesalpinioideae species are infrequent above 2500 m elevation in the tropics and are notably absent from mid- and high-elevation tropical montane forests, with only a few exceptions (e.g. some *Inga* Mill. spp., Paraseriantheslophantha(Vent.)I.C. Nielsensubsp.montana (Jungh.) I.C. Nielsen). The ecological versatility of the subfamily across the lowland tropical moisture availability spectrum is matched by its great diversity of life-history strategies, from massive canopy-emergent rainforest trees to small desert shrubs, and functionally-herbaceous savanna geoxyles to woody lianas and aquatic plants (Lewis et al. 2005; LPWG 2013, 2017; Koenen et al. 2020b; Ringelberg et al. 2022). Many species are economically important because of their highly-nutritious fruits, valuable wood, nitrogen-rich leaves and other products (Lewis et al. 2005) and are especially prominent as multipurpose trees in tropical silvo-pastoral and other agroforestry systems. Several other species constitute some of the world’s most serious invasive weeds (e.g. *Leucaenaleucocephala* (Lam.) de Wit, several *Mimosa* L. spp. and *Acacia* Mill. spp., *Prosopisjuliflora* (Sw.) DC.). Generic diversity is highest in the Neotropics and Africa and there are important centres of species diversity in Mexico and Central America, lowland South America, Africa, Madagascar, parts of S.E. Asia and Australia. Caesalpinioideae includes some of the largest genera in the legume family, such as *Acacia* with > 1,000 species concentrated in dry parts of Australia and *Mimosa* with > 500 species mostly in the Neotropics, as well as *Chamaecrista* Moench and *Senna* Mill., each with 300+ species distributed pantropically, *Inga* Mill. with ca. 300 species restricted to the Neotropics, almost entirely in rainforests and *Vachellia* Wight & Arn. (ca. 160 species) and *Senegalia* Raf. (ca. 220 species), two pantropical genera concentrated in drier environments, within which the iconic umbrella-crown trees of African savannas are found.

Numbers of genera across Caesalpinioideae have increased progressively through the last 270 years, but are difficult to track, because of the altered delimitation of the subfamily. However, the history of generic delimitation in mimosoids illustrates the overall trajectory of numbers of genera. Von Linnaeus (1753) placed all known mimosoids in a single genus *Mimosa*, which was later subdivided by Willdenow (1805) into five genera: *Inga*, *Mimosa*, *Schrankia* Willd., *Desmanthus* Willd. and *Acacia*. In 1825, de Candolle added five more genera, but the real foundations for all subsequent work were established by Bentham (1842, 1875) notably in his ‘Revision of suborder Mimoseae’ in 1875, which recognised six tribes and 46 genera, based on examination of 1,200 species known at that time.

**Figure 1.**
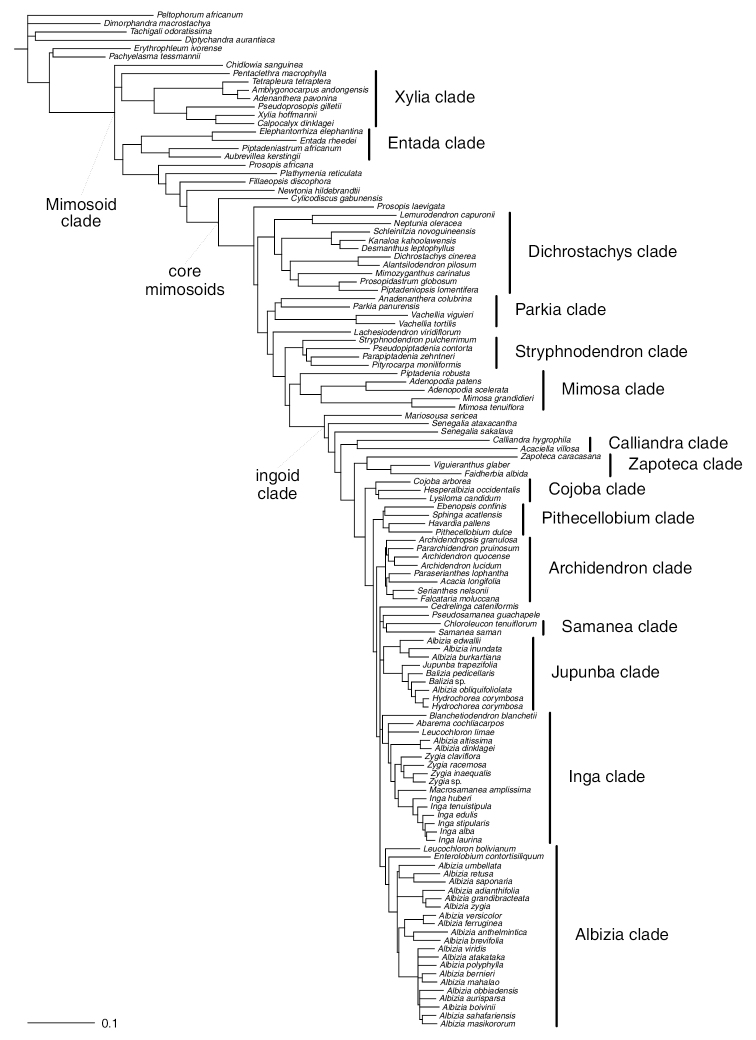
Phylogeny of Caesalpinioideae with clade names as inferred by Koenen et al. (2020b), the starting point for this study.

The legacy of Bentham’s generic system has been long-lasting. At the heart of Bentham’s system were a set of large, geographically widespread genera, including *Acacia*, *Calliandra* Benth., *Pithecellobium* Mart. and *Prosopis* L., all of which, with the advent of molecular phylogenetics, have been shown to be non-monophyletic. The disintegration of *Acacia* into (currently) seven segregate genera (*Acacia*, *Acaciella* Britton & Rose, *Mariosousa* Seigler & Ebinger, *Parasenegalia* Seigler & Ebinger, *Pseudosenegalia* Seigler & Ebinger, *Senegalia* and *Vachellia*), based on 20 years of molecular phylogenetic studies (Clarke et al. 2000; Miller and Bayer 2000, 2001, 2003; Robinson and Harris 2000; Luckow et al. 2003; Miller et al. 2003, 2013, 2017; Murphy et al. 2003; Seigler et al. 2006a, b; Brown et al. 2008; Bouchenak-Khelladi et al. 2010; Gómez-Acevedo et al. 2010; Miller and Seigler 2012; Kyalangalilwa et al. 2013; Mishler et al. 2014; Boatwright et al. 2015; Terra et al. 2017; Koenen et al. 2020b) (Figs 1 and 6–8) has been the most prominent example in legumes of the dissolution of one of Bentham’s broadly circumscribed pantropical genera. *Pithecellobium* and *Calliandra* have suffered similar fates (Barneby and Grimes 1996, 1997; Barneby 1998; de Souza et al. 2013, 2016). In contrast, although Bentham (1875) had restricted his concept of the genus *Albizia* Durazz. to just Old World species, Nielsen (1981) expanded the genus pantropically, creating the last big ‘dustbin genus’ of mimosoids (Koenen et al. 2020b). By far the most persistent generic delimitation problems surround those of former tribe Ingeae, where starkly contrasting generic systems and numerous generic transfers have caused much on-going confusion (reviewed by Brown 2008).

By 1981, the number of mimosoid genera had risen to 62 in *Advances in Legume Systematics* Part 1 (Elias 1981), 78 in *Legumes of the World* (Lewis et al. 2005) and in the most recent census (LPWG 2017) to 84, with 148 genera recognised in Caesalpinioideae as a whole.

Across the non-mimosoid Caesalpinioideae generic delimitation has also seen many changes. The most complex problems have been, without doubt, in the Caesalpinia Group and, especially, the genus *Caesalpinia* L. s.l. (Polhill and Vidal 1981; Lewis 1998; Gagnon et al. 2016), but these have now largely been resolved with the phylogenetically-based generic system of Gagnon et al. (2016), which recognised 26 genera, leaving just one residual generic problem in that group (see Clark et al. 2022).

Since LPWG (2017), two genera of Caesalpinioideae have been synonymised (i.e. *Cathormion* Hassk. within *Albizia* (Koenen et al. 2020b) and *Lemuropisum* H. Perrier within *Delonix* Raf. (Babineau and Bruneau 2017)) and six new genera have been segregated or resurrected (i.e. *Lachesiodendron* P.G. Ribeiro, L.P. Queiroz & Luckow (Ribeiro et al. 2018), *Parasenegalia* and *Pseudosenegalia* (Seigler et al. 2017), *Jupunba* Britton & Rose and *Punjuba* Britton & Rose (Soares et al. 2021) and *Robrichia* (Barneby & J.W. Grimes) A.R.M. Luz & E.R. Souza (de Souza et al. 2022a)), bringing the current tally of Caesalpinioideae genera to 152, of which 90 are mimosoids.

Despite this rapid on-going progress to align genera with clades in recent years, generic delimitation across Caesalpinioideae and, especially, the mimosoid clade, remains in a state of considerable flux and there is evidence to suggest that several more genera are non-monophyletic: *Prosopis* (Catalano et al. 2008), *Dichrostachys* (DC.) Wight & Arn. (Hughes et al. 2003; Luckow et al. 2005), *Balizia* Barneby & J.W. Grimes (Iganci et al. 2016; Koenen et al. 2020b), *Zygia* P. Browne (Ferm et al. 2019), *Entada* Adans. (Luckow et al. 2003), *Caesalpinia* (Gagnon et al. 2016), *Albizia*, *Senegalia* and *Leucochloron* Barneby & J.W. Grimes (Koenen et al. 2020b; Fig. 1). One factor that has undoubtedly contributed significantly to this widespread generic non-monophyly is the potentially pervasive homoplasy of multiple morphological characters previously used for generic delimitation, as well as reliance on only a few characters for delimiting taxa. This has led to tribes defined solely on stamen number and fusion into a staminal tube (Bentham 1875) and ‘fruit genera’, such as *Calliandra*, which was defined by Bentham (1875), based on its characteristic elastically dehiscent fruit. All mimosoid tribes and the genus *Calliandra* have since been shown to be non-monophyletic and their defining characters shown to have evolved multiple times across the subfamily (e.g. LPWG 2013; Barneby 1998). Such over-reliance on a small number of potentially homoplasious morphological characters, such as fruit type, connation and number of stamens and floral heteromorphy have likely repeatedly misled classification and resulted in widespread generic non-monophyly.

Another issue has been delimitation of the mimosoid clade with on-going uncertainties surrounding the inclusion or not of certain genera (Luckow et al. 2000, 2003; Manzanilla and Bruneau 2012). Although lacking valvate petals in bud (the putative synapomorphy of mimosoids), morphologically some members of the informal Dimorphandra group of Polhill and Vidal (1981) and Polhill (1994) show many similarities to mimosoids, with small, often numerous, regular flowers arranged in spikes or spiciform racemes, the hypanthium contracted, the anthers sagittate and introrse, the stamens becoming the most conspicuous and attractive part of the flower and pollen in tetrads in a few genera (*Diptychandra* Tul. and *Dinizia* Ducke) with possible affinities to the polyads that characterise many mimosoid lineages (Banks et al. 2010). These mimosoid-like features have prompted inclusion of some genera such as *Dinizia* in the mimosoid clade in the past (e.g. Burkart 1943; Luckow et al. 2000). Although none of these mimosoid-like genera has flowers with petals valvate in bud, previous molecular phylogenetic analyses have unexpectedly placed two Dimorphandra group genera in the mimosoid clade: *Chidlowia* Hoyle and *Sympetalandra* Stapf. The monospecific west African genus *Chidlowia* was placed with high support within the mimosoid clade in analyses based on few genetic markers (Manzanilla and Bruneau 2012; LPWG 2017), a result which was confirmed by the phylogenomic analyses of Koenen et al. (2020b; Fig. 1). The small Asian genus *Sympetalandra* was also recovered in the mimosoid clade in the *matK* tree of LPWG (2017), but was not sampled by Koenen et al. (2020b). Although support for the mimosoid clade is robust and the branch subtending that clade is long (Koenen et al. 2020b; Fig. 1), such that the monophyly of mimosoids is not in doubt, not all Caesalpinioideae genera have been included in phylogenomic analyses. By sampling widely and densely across Caesalpinioideae as a whole, we aim to further resolve which genera are placed in the mimosoid clade.

Several other issues have hindered a more complete understanding of the phylogeny and tribal / generic classification of subfamily Caesalpinioideae. First, the legacy of the traditional subfamily classification meant that taxon sampling in previous phylogenetic studies focused primarily on either old sense Caesalpinioideae (i.e. the grade subtending mimosoids (the ‘Caesalpinieae grade’ of Manzanilla and Bruneau 2012) of new sense Caesalpinioideae (Bruneau et al. 2008; Manzanilla and Bruneau 2012)), or on the mimosoid clade (e.g. Luckow et al. 2003, 2005; Koenen et al. 2020b). Few studies, apart from the family-wide analysis of plastid *matK* sequences (LPWG 2017), have sampled densely and widely across Caesalpinioideae as a whole. Second, several parts of the Caesalpinioideae phylogeny have been recalcitrant to phylogenetic resolution using traditional DNA sequence loci, most notably along the backbone of the grade subtending the mimosoid clade (Bruneau et al. 2008; Manzanilla and Bruneau 2012; LPWG 2017) and across the large ingoid clade sensu Koenen et al. (2020b). Third, lack of dense pantropical sampling of taxa in previous phylogenies means that the monophyly of several key genera with wide pantropical distributions, such as the ‘dustbin genus’ *Albizia*, has not been adequately tested and that possible sister-group relationships between New and Old World groups that are relevant to delimitation of genera may have been missed.

More robust foundations to overcome these difficulties were established by Koenen et al. (2020b) in a phylogenomic study of the mimosoid clade. By developing a clade-specific bait set (*Mimobaits*) for targeted enrichment of 964 nuclear genes, Koenen et al. (2020b) opened the way for generating DNA sequence datasets orders of magnitude larger than those used previously, thereby providing much enhanced phylogenetic resolution. Using these new data, Koenen et al. (2020b) established a new phylogenomic framework and recognised three large informally named higher-level clades each successively nested within Caesalpinioideae (Fig. 1). The mimosoid clade, core mimosoid clade and ingoid clade were all strongly supported by high proportions of gene trees and subtended by long branches. In addition, a set of 15 smaller informally named subclades across mimosoids were proposed by Koenen et al. (2020b) (Fig. 1) to replace the previously defined tribes and informal groups and alliances, almost all of which have been shown by numerous studies to be non-monophyletic (Luckow et al. 2003; LPWG 2013, 2017; Koenen et al. 2020b). Furthermore, although the *Mimobaits* bait set was designed based on RNA-seq data from species of four mimosoid genera and used initially for the mimosoid clade, the results of Koenen et al. (2020b) suggested that they work well across the non-mimosoid Caesalpinioideae, opening the way to potentially sequence these genes across the subfamily as a whole. The Koenen et al. (2020b) study also further revealed or confirmed the non-monophyly of several genera, but it lacked sufficient taxon sampling to fully test generic monophyly and sampling was largely restricted to the mimosoid clade. Here, we capitalise on these foundations using a slightly modified version of the *Mimobaits* gene set covering 997 nuclear genes to extend taxon sampling to 420 species from 147 of the 152 genera and establish a robust phylogenomic hypothesis for subfamily Caesalpinioideae as a whole.

This new phylogeny provides the basis for testing the monophyly of genera (the main focus of this paper and of this Special Issue *Advances in Legume Systematics* (ALS) 14, Part 1), establishing a new higher-level classification of the subfamily (the focus of ALS 14, Part 2) and for downstream analyses of biogeography, trait evolution and diversification (de Faria et al. 2022; Ringelberg et al. 2022). Caesalpinioideae provides an excellent clade for investigating evolutionary diversification and phylogenetic turnover across the lowland tropics (Lavin et al. 2004; Gagnon et al. 2019; Ringelberg et al. 2020, 2022), as well as the evolution of several prominent plant functional traits including compound leaves, armature, extrafloral nectaries and ant associations (Marazzi et al. 2019), agglomeration of pollen into polyads, plant growth forms (Gagnon et al. 2019), floral morphology and pollination syndromes, fruit morphology and seed dispersal syndromes and the ability to form nitrogen-fixing root nodule symbiosis (Sprent et al. 2017; de Faria et al. 2022). However, all of these opportunities require a robust and well-sampled subfamily-wide phylogeny of Caesalpinioideae. In turn, some of these traits have been used for generic delimitation in the past and, in this paper, we also evaluate a handful of such traits in a preliminary way by mapping them on to the phylogeny.

## ﻿Methods

### ﻿Phylogeny: taxon and gene sampling, and tree building

To test generic monophyly as thoroughly as possible, we sampled taxa to encompass known or suspected cases of generic non-monophyly, as well as sets of representative species spanning the root nodes of larger genera in Caesalpinioideae (Suppl. material 1). The final phylogenomic dataset comprised 420 Caesalpinioideae taxa covering 147 of the 152 genera. The five missing genera are: *Stenodrepanum* Harms, the monospecific sister genus of *Hoffmannseggia* Cav. in the Caesalpinia Group (Gagnon et al. 2016); *Hultholia* Gagnon & G.P. Lewis, another monospecific genus in the Caesalpinia Group (Gagnon et al. 2016); *Microlobius* C. Presl, which is also monospecific and nested within the mimosoid genus *Stryphnodendron* Mart. (Simon et al. 2016; Ribeiro et al. 2018; Lima et al. 2022); *Vouacapoua* Aubl., a genus of three species, whose phylogenetic placement is uncertain, but most likely falls into the Cassia clade (Bruneau et al. 2008; LPWG 2017); and *Pterogyne* Tul., another monospecific genus whose placement has been uncertain (Manzanilla and Bruneau 2012; Zhang et al. 2020), but which is probably sister to all Caesalpinioideae, excluding the Arcoa and Umtiza clades (Zhao et al. 2021). In total, 89 of 90 mimosoid genera and 58 of the 62 non-mimosoid Caesalpinioideae genera were sampled.

**Figure 2.**
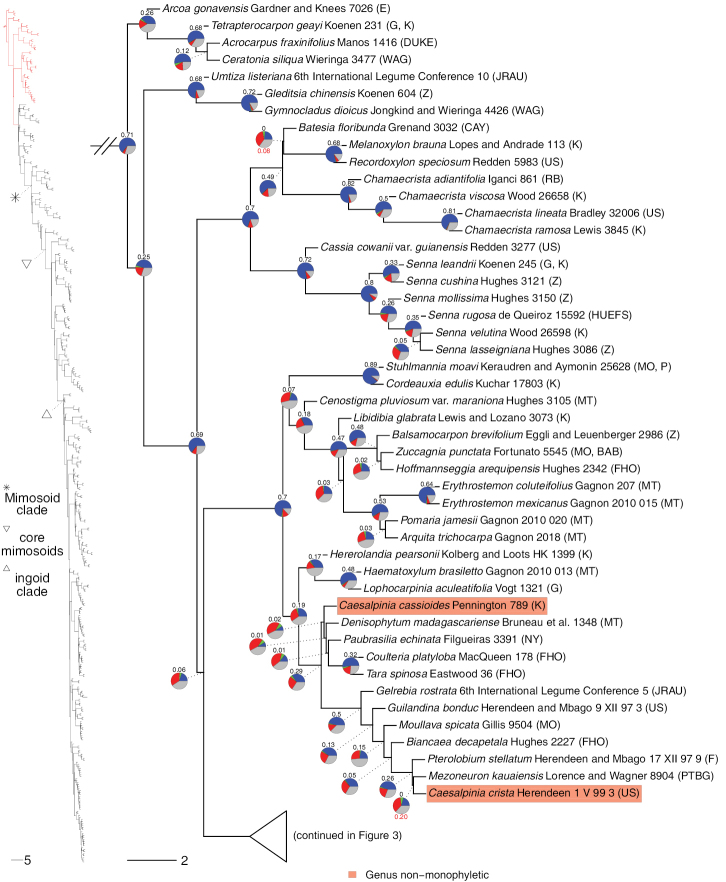
Phylogeny of Caesalpinioideae, part 1 (continued in Figs 3–12). Left part of figure shows complete Caesalpinioideae phylogeny with highlighted in red the part shown in detail on the right. Depicted phylogeny is the ASTRAL (Zhang et al. 2018) phylogeny based on 821 single-copy nuclear gene trees, with branch lengths expressed in coalescent units and terminal branches assigned an arbitrary uniform length for visual clarity. Genera resolved as (potentially) non-monophyletic are highlighted and clades recognised by Koenen et al. (2020b) are labelled. Support for relationships is based on gene tree conflict: pie charts show the fractions of supporting and conflicting gene trees per node calculated using PhyParts (Smith et al. 2015), with blue representing supporting gene trees, green gene trees supporting the most common alternative topology, red gene trees supporting further alternative topologies and grey gene trees uninformative for this node. Numbers above nodes are Extended Quadripartition Internode Certainty scores calculated with QuartetScores (Zhou et al. 2020). Numbers below nodes are the outcome of ASTRAL’s polytomy test (Sayyari and Mirarab 2018), which tests for each node whether the polytomy null model can be rejected. Only non-significant (i.e. > 0.05) scores are shown, i.e. only for nodes that are better regarded as polytomies according to the test.

**Figure 3.**
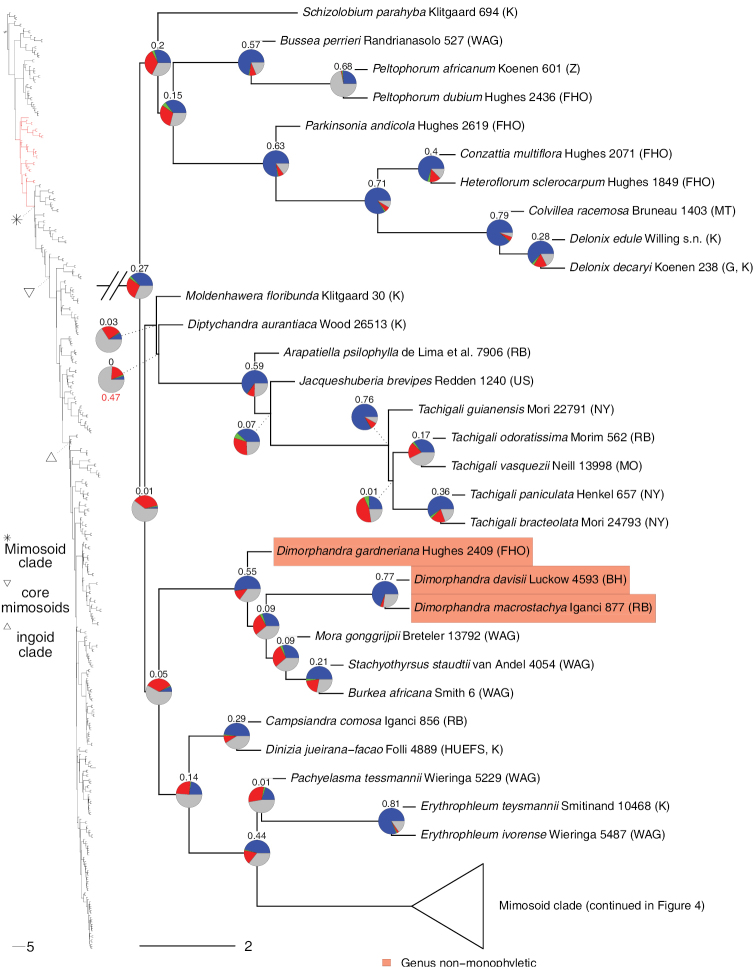
Phylogeny of Caesalpinioideae (continued). See Figure 2 for caption.

**Figure 4.**
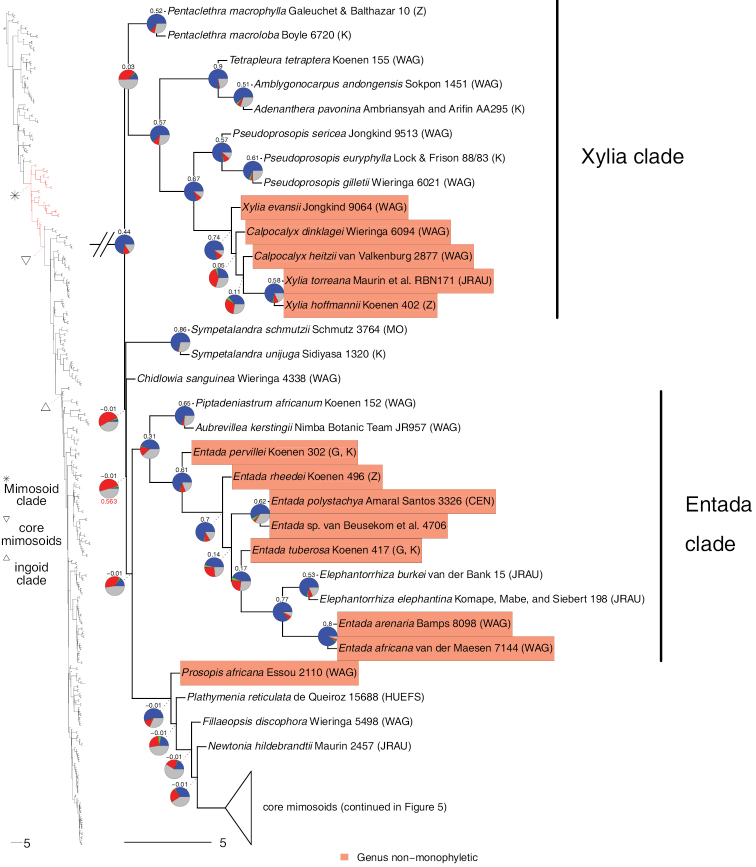
Phylogeny of Caesalpinioideae (continued). See Fig. 2 for caption.

**Figure 5.**
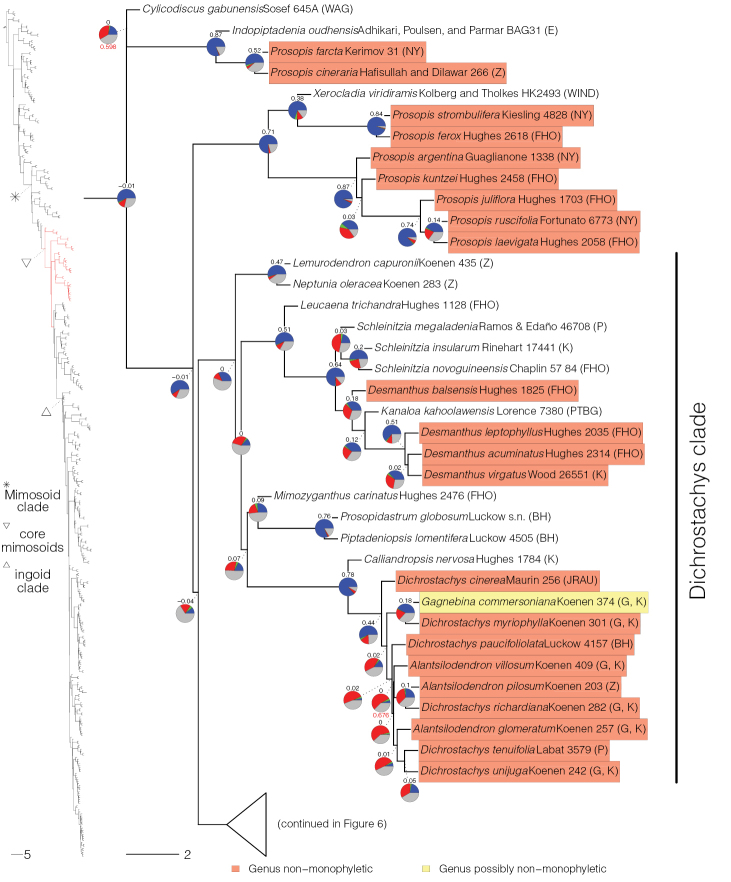
Phylogeny of Caesalpinioideae (continued). See Fig. 2 for caption.

**Figure 6.**
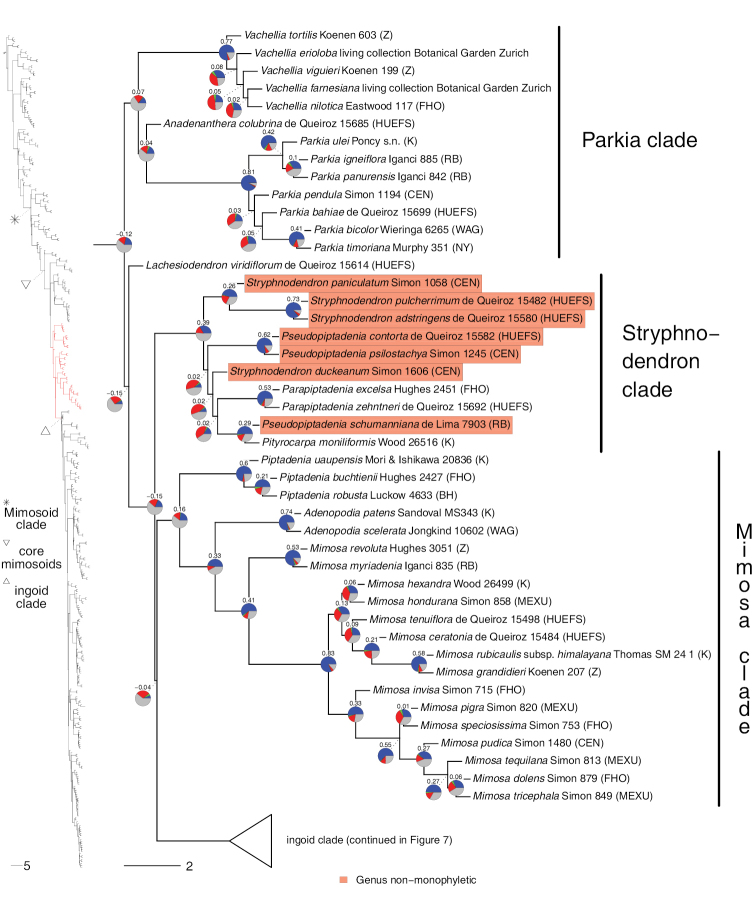
Phylogeny of Caesalpinioideae (continued). See Fig. 2 for caption.

**Figure 7.**
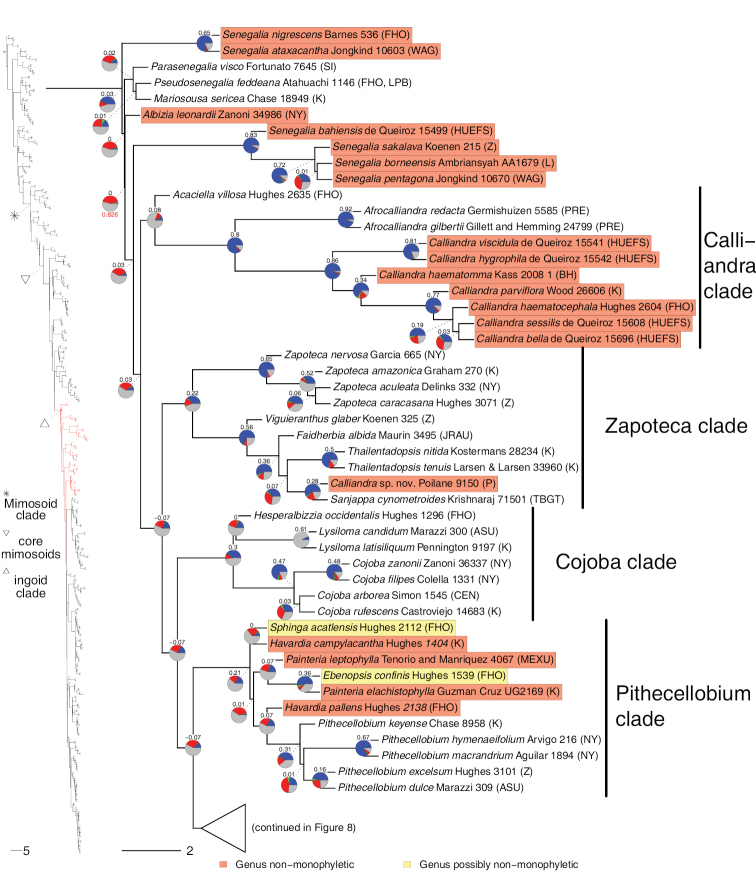
Phylogeny of Caesalpinioideae (continued). See Fig. 2 for caption.

**Figure 8.**
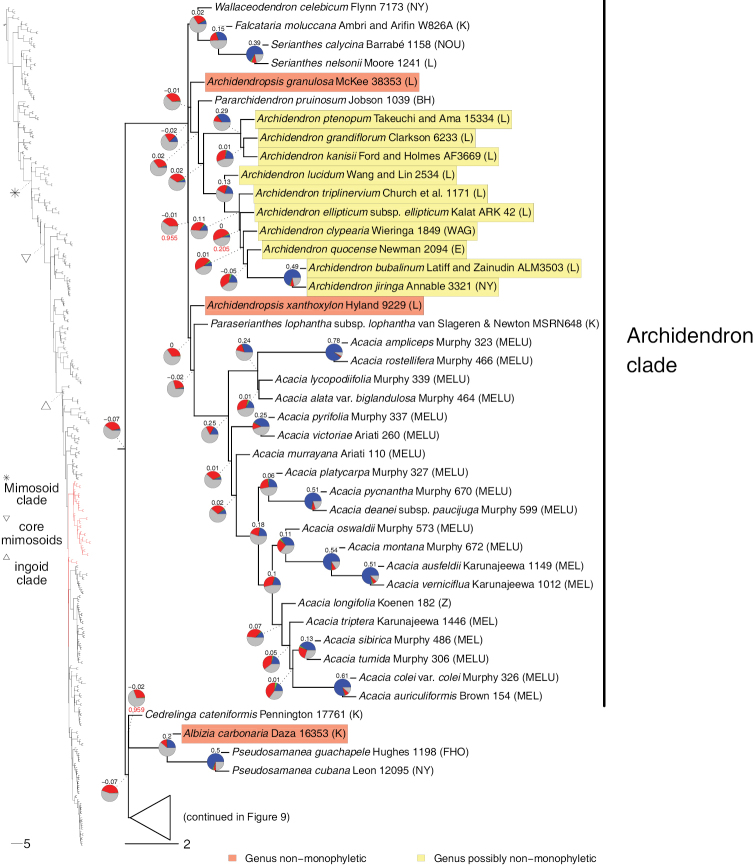
Phylogeny of Caesalpinioideae (continued). See Fig. 2 for caption.

**Figure 9.**
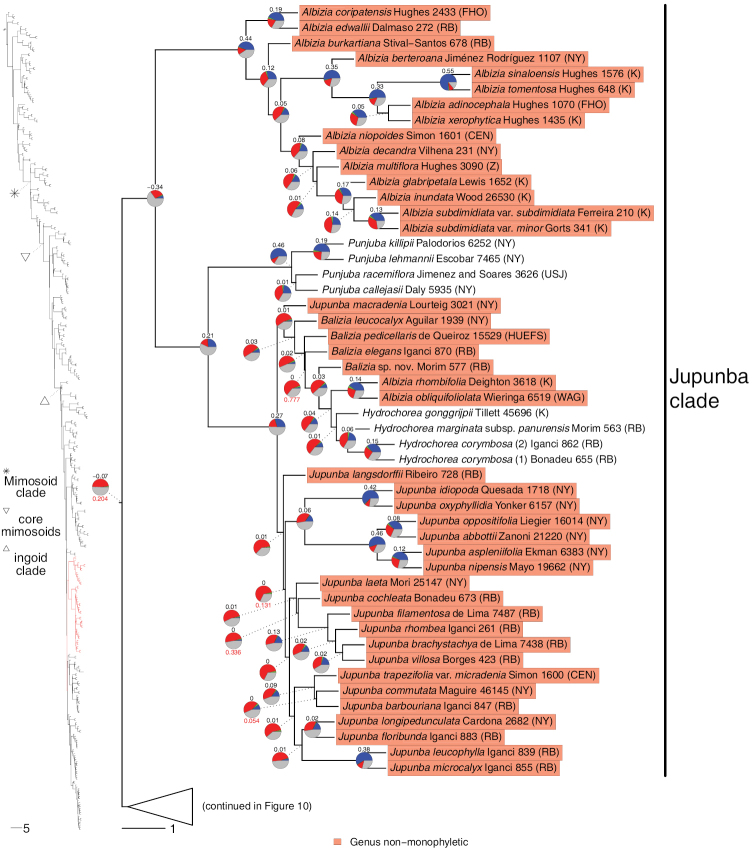
Phylogeny of Caesalpinioideae (continued). See Fig. 2 for caption.

**Figure 10.**
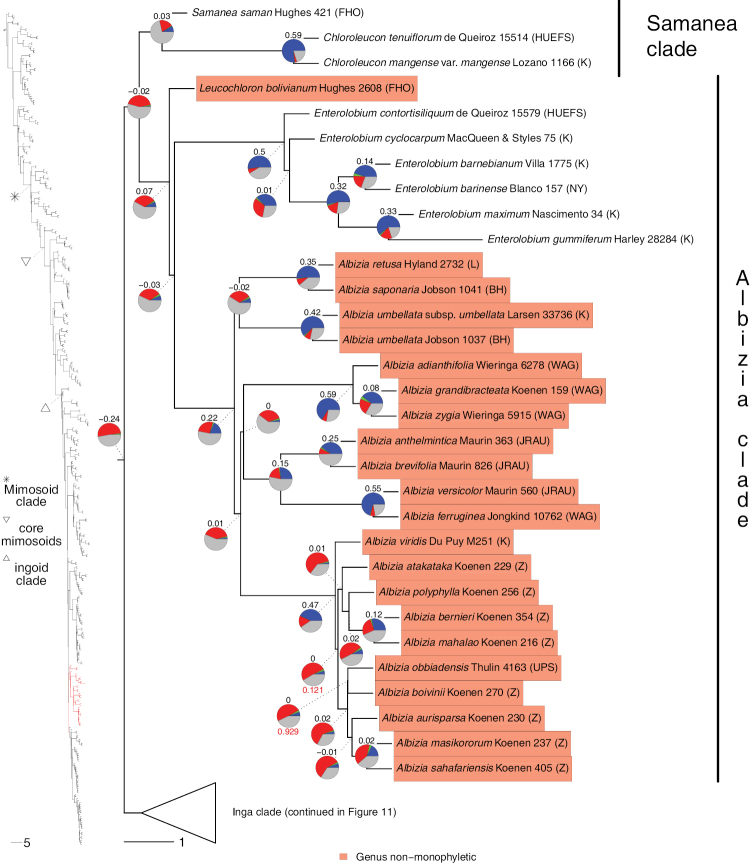
Phylogeny of Caesalpinioideae (continued). See Fig. 2 for caption.

**Figure 11.**
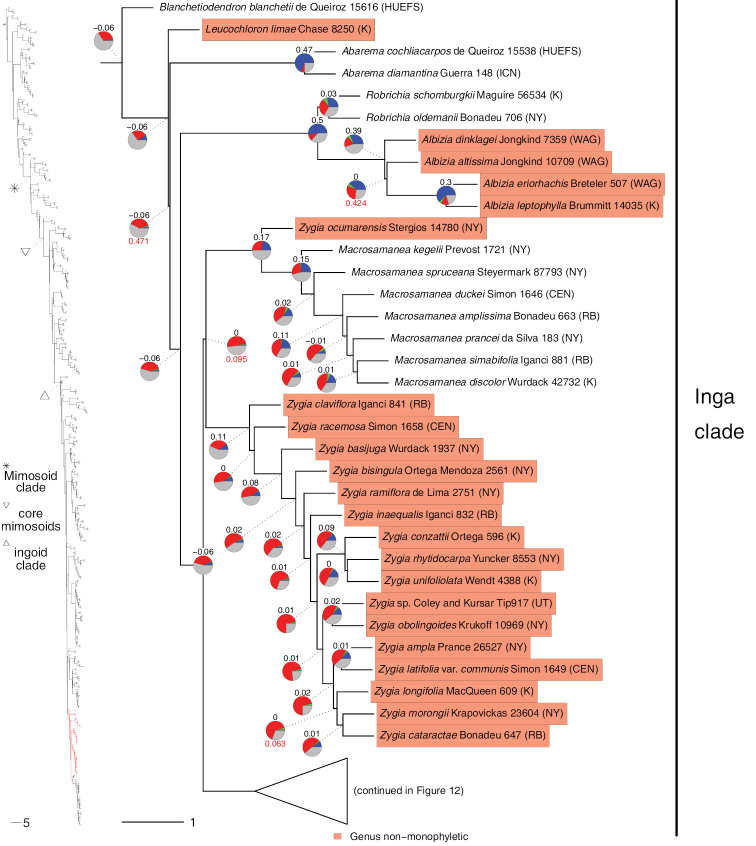
Phylogeny of Caesalpinioideae (continued). See Fig. 2 for caption.

**Figure 12.**
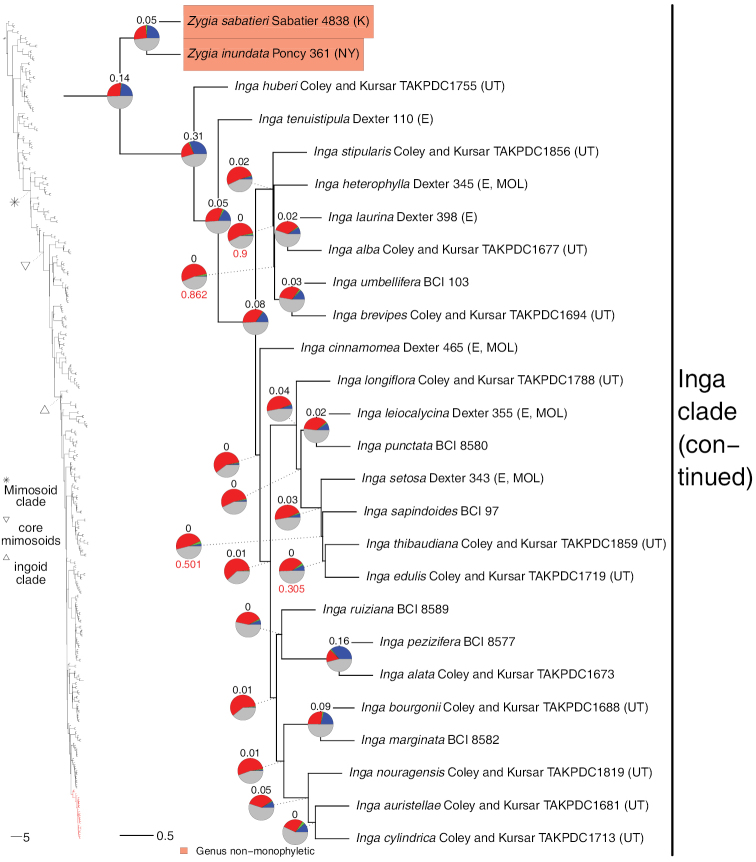
Phylogeny of Caesalpinioideae (continued). See Fig. 2 for caption.

We sequenced a set of 997 nuclear genes specifically selected for phylogenomic analyses of the mimosoid clade (Koenen et al. 2020b) via targeted enrichment and hybrid capture. This Hybseq approach has quickly become the method of choice to generate phylogenomic data because of its versatility and relatively low cost (e.g. Nicholls et al. 2015; Barrett et al. 2016; Hart et al. 2016; Dodsworth et al. 2019; Johnson et al. 2019; Koenen et al. 2020b). Library preparation, hybrid capture, enrichment and sequencing were performed by Arbor Biosciences (previously MYcroarray; Ann Arbor, USA). Full details about how the new Caesalpinioideae phylogeny was inferred are presented by Ringelberg et al. (2022), but briefly, HybPiper (Johnson et al. 2016) was used to assemble the loci and the pipeline of Yang and Smith (2014) was used for data cleaning and orthology assessment. Various phylogenetic methods, including the multi-species coalescent approach using individual gene trees with ASTRAL (Zhang et al. 2018), Maximum Likelihood based on concatenated alignments with RAxML (Stamatakis 2014) and Bayesian gene jack-knifing with PhyloBayes (Lartillot et al. 2013), were used to infer ten nuclear species trees, which also differ in whether nucleotide or amino acid sequences were used and in the way orthology was assessed (Ringelberg et al. 2022). In addition, a chloroplast phylogeny was inferred using off-target plastid sequences, bringing the total number of phylogenies to eleven. Topological congruence between these eleven different phylogenies was assessed. Support for relationships was expressed in numbers of supporting and conflicting gene trees using PhyParts (Smith et al. 2015) and QuartetScores (Zhou et al. 2020) (Figs 2–12), rather than conventional bootstrap or posterior support values that are known to be inflated in large phylogenomic datasets (Rokas and Carroll 2006; Pease et al. 2018).

### ﻿Character evolution

To explore evolution of morphological traits that have been important for generic delimitation, we scored variation in armature, aspects of floral heteromorphy and mode of fruit dehiscence and mapped their distribution across the Caesalpinioideae phylogeny. Our goal was to highlight how an over-reliance on broadly-defined character complexes or functional traits may have misled classification in the past, rather than to perform detailed reconstructions of character evolution through time or to thoroughly assess the homology of various character states.

The three character complexes and their states were defined as follows:

- armature (six states): unarmed; nodal or internodal prickles on stem; stipular spines; nodal axillary thorns, including the axillary inflorescence axes which are modified into spines in *Chloroleucon* (Benth.) Britton & Rose; spinescent shoots.
- floral heteromorphy (three states): homomorphic, i.e. with no conspicuous modification or variation amongst flowers within an inflorescence (here we include inflorescences that do not show any conspicuous phenotypic variation beyond the very common occurrence of variable proportions of male and bisexual flowers within inflorescences of many mimosoid genera); heteromorphic 1 = basal flowers of the inflorescence with showy staminodia; heteromorphic 2 = the central flower (or flowers) enlarged/sessile cf. the peripheral (sometimes pedicellate) flowers.
- pod dehiscence (six states): indehiscent; inertly dehiscent along one or both sutures; explosively dehiscent, the woody valves twisting and splitting along both sutures along whole length of pod simultaneously; elastically dehiscent from the apex, the valves recurving, but not laterally twisting; craspedium, fruits breaking up into free-falling one-seeded articles leaving a persistent replum or whole valve breaking away intact from replum (valvately dehiscent); lomentiform fruit, the valves readily cracking between the seeds into one-seeded articles, taken here to include crypto-lomentiform fruits.

Data were assembled from taxonomic monographs, revisions and floras. Character evolution was simulated across the phylogeny using the ‘make.simmap’ function in the phytools (Revell 2012) R (R Core Team 2022) package, with 300 independent simulations and a ‘symmetrical rates’ (SYM) model. In each analysis, the character complex of interest (i.e. armature, floral heteromorphy and pod dehiscence) was treated as a single character with multiple states. A rooted phylogeny, without outgroups, was used for the analyses. The root character state was assigned an uninformed prior (i.e. each character state had the same initial probability of occurrence).

### ﻿Data availability

A tree file of the ASTRAL phylogeny based on the single-copy genes (depicted in Figs 2–12) is included as online Suppl. material 4. In this tree file, all taxon names have been updated to reflect taxonomic changes made in all the entries in Advances in Legume Systematics 14 Part 1.

## ﻿Results

### ﻿Phylogenomics

For full results of the sequencing, orthology assembly and phylogenetic inference, see Ringelberg et al. (2022). Here a brief overview is provided.

Hybrid capture and sequencing yielded a large phylogenomic dataset with little missing data: the concatenated nucleotide alignment of the 821 single-copy nuclear genes (a subset of all 997 genes, see below) contains 944,871 sites, 824,713 alignment patterns (i.e. an indication of the phylogenetic informativeness of the alignment, determined by RAxML) and only 11.88% gaps. The ten nuclear species trees that were inferred using different phylogenetic methods are well-supported in terms of gene tree congruence measures (Figs 2–12) and largely congruent with each other. The few topological differences between different phylogenies typically involve only small numbers of species within relatively recent radiations, or deeper putative polytomies such as along the backbone of the ingoid clade, characterised by lack of phylogenetic signal across almost all genes (Koenen et al. 2020b), or the backbone of the Archidendron clade (Fig. 8), characterised by both lack of signal and high conflict amongst gene trees. These minor topological differences do not affect any of the findings of generic non-monophyly discussed below.

The plastid phylogeny (Suppl. material 3) differs more substantially from the nuclear species trees, reflecting the fact that nuclear and chloroplast genomes have unique and sometimes conflicting evolutionary histories (Bruun-Lund et al. 2017; Lee-Yaw et al. 2019; Rose et al. 2021). Cytonuclear discordance affects the monophyly of *Senegalia* Raf. (Terra et al. 2022), *Archidendron* F. Muell. (Brown et al. 2022), *Dimorphandra* Schott, the placement of *Desmanthusbalsensis* J.L. Contreras (Hughes et al. 2022b) and whether *Zygiainundata* (Ducke) H.C. Lima ex Barneby & J.W. Grimes and *Z.sabatieri* Barneby & J.W. Grimes form the sister clade of *Inga* or a grade subtending *Inga*.

Hereafter the ASTRAL phylogeny based on the subset of 821 single-copy nuclear gene trees is used as the ‘reference’ Caesalpinioideae backbone phylogeny (Figs 2–12). We use this particular tree over the plastome phylogeny because the nuclear dataset is based on hundreds of independent loci and contains considerably more sites, taxa and fewer gaps, while the plastome phylogeny is based on a single non-recombining locus. The nuclear trees, therefore, likely better represent an approximation of the true evolutionary history of Caesalpinioideae than the phylogeny based on maternally inherited plastid data. Of the various nuclear trees, we select the ASTRAL phylogeny because we find extensive conflict amongst individual gene trees in certain parts of the phylogeny (Figs 2–12), which violates the central assumption of the concatenation model (Jiang et al. 2020) and because the multi-species coalescent model has been shown to consistently outperform the concatenation model on a range of phylogenomic datasets (Jiang et al. 2020). Our analyses reveal that different approaches to orthology assessment have a very minor impact on the final Caesalpinioideae phylogeny, likely because the vast majority of nuclear genes in our dataset are single-copy (i.e. 821 of 997) (see Ringelberg et al. 2022 for details). Nevertheless, how to deal with multi-copy genes is a contentious topic in phylogenetics (Yang and Smith 2014; Moore et al. 2018; Karimi et al. 2019) and we, therefore, focus on the ASTRAL phylogeny based on just the 821 single-copy genes.

The resultant ASTRAL phylogeny is, in general, robustly supported across the majority of nodes using measures of gene tree support and conflict (Figs 2–12). However, there are also some specific parts of the phylogeny which show high levels of gene tree conflict and/or lack of phylogenetic signal across large fractions of genes, which appears to be a feature of most phylogenies based on large phylogenomic datasets (Salichos and Rokas 2013; Wang et al. 2019; Jiang et al. 2020; Koenen et al. 2020a,b; Yang et al. 2020). In most cases, the primary source of gene tree conflict is limited signal in individual gene trees rather than the presence of strongly-supported alternative topologies amongst the gene trees (Figs 2–12, Koenen et al. 2020b), suggesting that the conflict often has methodological rather than biological causes and implying that the presence of conflict per se is no reason for doubts about the recovered Caesalpinioideae topology. However, some parts of the phylogeny with high levels of gene tree conflict or lack of signal may be better viewed as potential polytomies, including the previously identified putative hard polytomy subtending a set of six or seven lineages along the backbone of the ingoid clade (Koenen et al. 2020b) and a putative polytomy across the backbone of the large Archidendron clade (see Appendix 1). These parts of the phylogeny showing high gene tree conflict affect only a few decisions about generic delimitation, most notably across the grade comprising *Senegalia* and allies (Fig. 7; Terra et al. 2022) and across the backbone of the Archidendron clade (Fig. 8; Brown et al. 2022).

All the informally named clades of Koenen et al. (2020b; Fig. 1) are here confirmed with robust support in this new phylogeny (Figs 2–12), including the mimosoid clade that is robustly supported and subtended by a long branch (Fig. 4). Our results confirm placement of *Chidlowia* and *Sympetalandra* within the mimosoid clade and *Dinizia* outside the mimosoid clade, with high support (Fig. 4). Higher-level relationships that form the basis for the clade- and tribal-based classification of Caesalpinioideae presented in “Advances in Legume Systematics 14, Part 2”, are not further discussed here.

### ﻿Generic non-monophyly

Twenty-two genera were recovered as non-monophyletic or were nested within another genus and, therefore, likely require generic re-delimitation (Figs 2–12; Appendix 1). In addition, based on our results, the taxonomic status of *Gagnebina* Neck. ex DC., *Sphinga* Barneby & J.W. Grimes and *Ebenopsis* Britton & Rose, each represented here by a single taxon and nested in clades with complex generic relationships, require additional species sampling. Furthermore, although *Archidendron* species form a clade (Fig. 8), the genus is not supported as monophyletic in a substantial fraction of the individual gene trees (Fig. 8), nor in the plastid tree (Suppl. material 3) (see Brown et al. 2022). Overall, our results therefore show that 14(–17)% of the 152 Caesalpinioideae genera require re-delimitation and taxonomic updating. Only two of these genera are non-mimosoid Caesalpinioideae: *Dimorphandra* Schott and *Caesalpinia*. Almost all the non-monophyly issues are, therefore, in the mimosoid clade, where 22(–27)% of the 90 genera will require name changes.

Appendix 1 lists all (potentially) non-monophyletic genera with notes and pointers to papers in this Special Issue that discuss these genera and, in many cases, propose nomenclatural changes that resolve many of the non-monophyly issues revealed in our analyses. In some cases, it is clear that formal taxonomic re-circumscription must await more densely-sampled phylogenies and detailed morphological analyses. It is also important to note that, unless explicitly stated otherwise, the reported generic non-monophyly is recovered in all trees (i.e. the nuclear ASTRAL, RAxML and PhyloBayes species trees and chloroplast phylogeny) with high support values expressed and assessed in terms of numbers or fractions of supporting or conflicting genes.

### ﻿Character evolution

**Figure 13.**
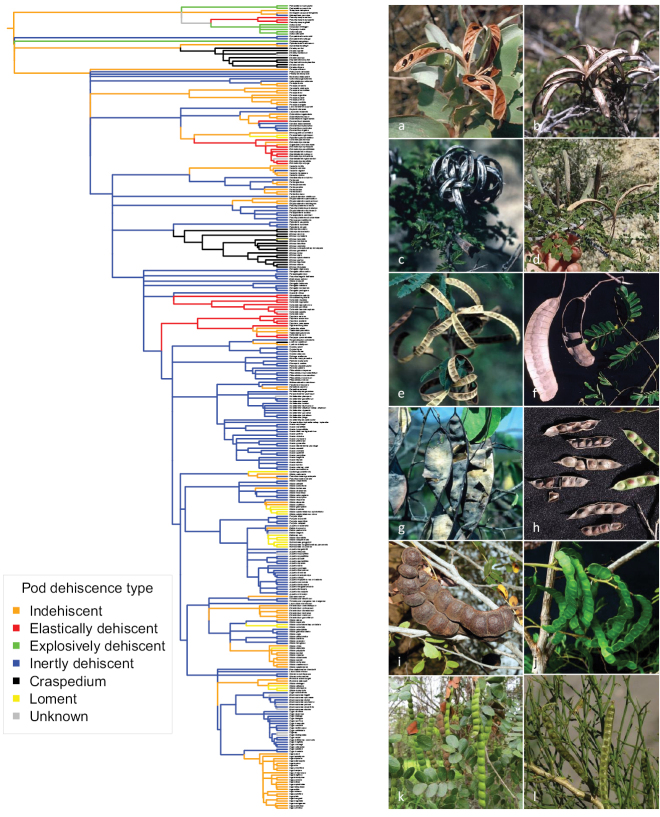
Evolution of fruit dehiscence types across the mimosoid clade. Character states were defined as: *indehiscent*; *inertly dehiscent* along one or both sutures; *explosively dehiscent*, whereby the woody valves twist and split along both sutures along whole length of pod simultaneously; *elastically dehiscent* from the apex, the valves recurving, but not laterally twisting; *craspedium*, i.e. fruits breaking up into free-falling one-seeded articles leaving a persistent replum or whole valve breaking away intact from replum (valvately dehiscent); *lomentiform* fruit, i.e. the valves readily cracking between the seeds into one-seeded articles, taken here to include crypto-lomentiform fruits. Branch lengths are not informative in this figure. Photos **a–e** elastically dehiscent **a***Acaciaargyraea* Tindale **b***Calliandraprostrata* Benth. **c***Calliandropsisnervosa* (Britton & Rose) H.M. Hern. & P. Guinet **d***Alantsilodendronmahafalense* (R. Vig.) Villiers **e***Zapotecaportoricensis* (Jacq.) H.M. Hern **f–h** craspedium **f***Entadapolystachya* (L.) DC. **g***Lysilomatergeminum* Benth. **h**MimosamontanaKunth.var.sandemanii Barneby **i–l** lomentiform **i***Albiziamoniliformis* (DC.) F. Muell. **j***Albiziasubdimidiata* (Splitg.) Barneby & J.W. Grimes **k***Albiziapistaciifolia* (Willd.) Barneby & J.W. Grimes **l***Prosopidastrumglobosum* (Gillies ex Hook. & Arn.) Burkart. Photos **a** Bruce Maslin **b, c, e–h** Colin Hughes **d**http://clubbotatoliara.e-monsite.com/pages/posters-films-rapports/photos.html**i** Garry Sankowsky http://www.rainforestmagic.com.au**j** Marcelo Simon **k** Xavier Cornejo **l**https://www.floramendocina.com.ar.

**Figure 14.**
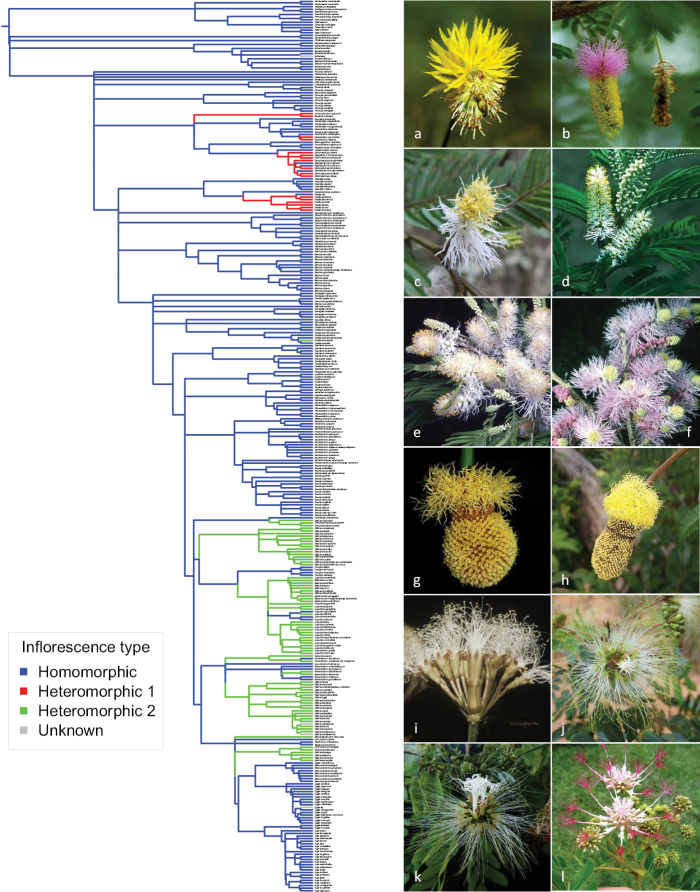
Evolution of types of floral heteromorphy across the mimosoid clade. Character states were defined as: *homomorphic*, i.e. with no conspicuous modification or variation amongst flowers within an inflorescence (here we include inflorescences that can comprise proportions of male and bisexual flowers, but no other more conspicuous variation); *heteromorphic 1* = basal flowers of the inflorescence with showy staminodia; *heteromorphic 2* = flowers dimorphic within an inflorescence, the central flower (or flowers) enlarged/sessile cf. the peripheral (sometimes pedicellate) flowers. Branch lengths are not informative in this figure. Photos **a–h** heteromorphic 1 **a***Neptuniaplena* (L.) Benth. **b***Dichrostachyscinerea* (L.) Wight & Arn. **c***Dichrostachysmyriophylla* Baker **d***Gagnebinapterocarpa* (Lam.) Baill. **e***Dichrostachysbernieriana* Baill. **f***Dichrostachysakataensis* Villiers **g***Parkiabahiae* H.C. Hopkins **h***Parkianitida* Miq. **i–l** heteromorphic 2 **i***Pseudosamaneaguachapele* (Kunth) Harms **j***Albiziaobliquifoliolata* De Wild. **k***Hydrochoreacorymbosa* (Rich.) Barneby & J.W. Grimes **l***Albiziagrandibracteata* Taub. Photos **a, b, g, i** Colin Hughes **c, k, l** Erik Koenen **d** Melissa Luckow **e, f** Dave Du Puy **h** Giacomo Sellan https://identify.plantnet.org/the-plant-list/observations/1012799991**j** Jan Wieringa.

**Figure 15.**
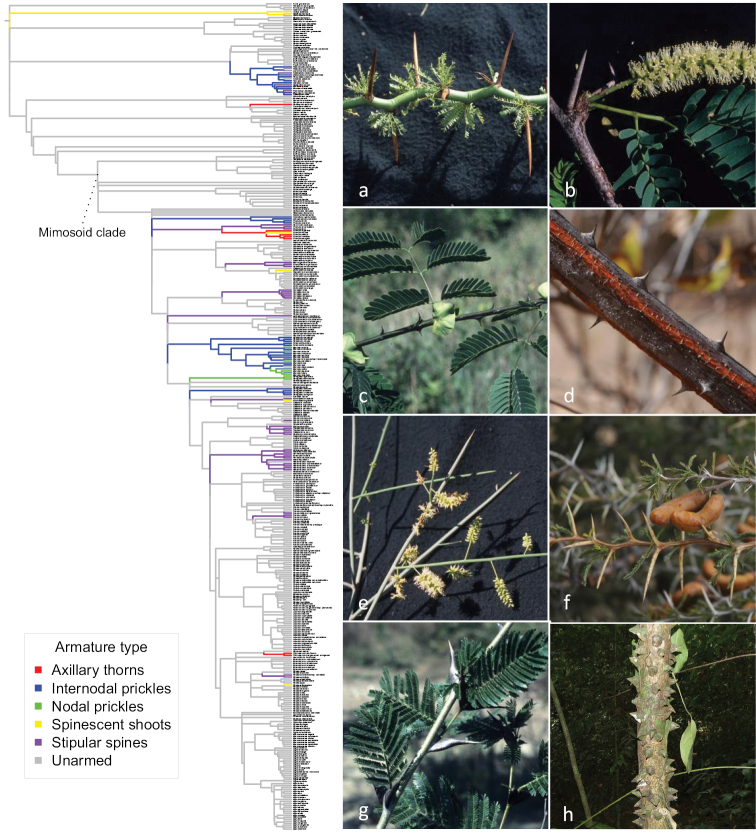
Evolution of different types of armature across Caesalpinioideae. Character states were defined as: *unarmed*; *nodal or internodal prickles on stem*; *stipular spines*; *nodal axillary thorns* including modified inflorescence axes of *Chloroleucon*; *spinescent shoots*. Branch lengths are not informative in this figure. Photos **a** and **b** axillary thorns **a***Parkinsoniaandicola* (Griseb.) Varjão & Mansano **b***Prosopisjuliflora* (Sw.) DC. **c, d, h** internodal prickles **c***Senegaliatamarindifolia* (L.) Britton & Rose **d***Mimosaophthalmocentra* Mart. ex Benth. **e** spinescent shoots, *Prosopiskuntzei* Harms **f** and **g** stipular spines **f***Prosopisferox* Griseb. **g***Vachelliacornigera* (L.) Seigler & Ebinger **h***Cylicodiscusgabunensis* Harms. All photos Colin Hughes, except **h** William Hawthorne.

Armature, types of inflorescence heteromorphy and pod dehiscence type each show high levels of homoplasy (Figs 13–15, Table S2) with all types of armature, floral heteromorphy and pod dehiscence hypothesised to have evolved multiple times.

## ﻿Discussion

### ﻿Generic non-monophyly

The new Caesalpinioideae phylogeny (Figs 2–12) reveals extensive generic non-monophyly: 22 genera are non-monophyletic or nested within another genus and four other genera could likely also be non-monophyletic (Appendix 1). Notably, there are just two non-monophyletic genera (3% of the 62) across the non-mimosoid Caesalpinioideae, while 20 (to 24) mimosoid genera (i.e. 22(–27)% of 90 genera) are non-monophyletic. The discovery of such a high level of generic non-monophyly in the mimosoid clade is likely attributable to the denser taxon sampling in mimosoids than non-mimosoids in our analyses; the greater species-richness of mimosoids, which account for ca. 75% of the ca. 4,600 Caesalpinioideae species (LPWG 2017), but only 59% of the 152 genera, indicating that, on average, mimosoid genera are more species-rich and, therefore, more likely to have monophyly issues than non-mimosoid Caesalpinioideae genera; the fact that the Caesalpinia Group, the most problematic clade of non-mimosoid Caesalpinioideae in terms of generic delimitation, was already largely resolved by Gagnon et al. (2016), further reducing the likelihood of non-monophyly issues across non-mimosoid Caesalpinioideae; and finally, the continued legacy of Bentham’s broadly circumscribed mimosoid genera which has still not been fully resolved. For example, *Acacia*, which as indicated earlier, was once a pantropical genus with over 1,400 species (Miller and Seigler 2012) and now comprises seven genera, yet one of these genera, *Senegalia*, is here recovered as non-monophyletic (Fig. 7) and further subdivision of *Senegalia* seems likely (Terra et al. 2022). Similarly, *Calliandra* once had a pantropical distribution until Barneby (1998) restricted it to the New World (de Souza et al. 2013). However, not all Old World *Calliandra* species have yet been assigned to other genera and *Calliandra*, therefore, also remains non-monophyletic (Fig. 7). Finally, *Albizia*, the last mimosoid ‘dustbin genus’ (Barneby and Grimes 1996; Brown 2008; Koenen et al. 2020b) is here confirmed to be non-monophyletic in line with previous findings (Koenen et al. 2020b) (Figs 7–11), but with two previously unsampled Neotropical species each representing additional evolutionary lineages (Terra et al. 2022; Koenen 2022b). Nevertheless, most African, Madagascan and Asian *Albizia* species do form a single clade (Fig. 10; Koenen et al., unpublished data), while most Neotropical species are also in a single clade (Aviles et al. 2022) (Fig. 9, see Appendix 1).

### ﻿Morphological homoplasy

Given the extensive re-arrangements of genera in Caesalpinioideae over the last two decades, the question arises why such a significant fraction of genera is still non-monophyletic in these new phylogenomic analyses. We identify two main reasons for this. First, extensive morphological homoplasy has misled generic delimitation and second, lack of pantropical taxonomic synthesis and phylogenetic sampling have resulted in failure to identify clades that span the Old World and New World or, conversely, amphi-Atlantic genera that are non-monophyletic, i.e. potential trans-continental connections and disconnects.

First, and most importantly, the likely extent of homoplasy of morphology and functional traits across Caesalpinioideae is only now starting to be revealed using this new phylogeny (Figs 13–15; de Faria et al. 2022). Here, we reconstructed hypotheses for the evolutionary trajectories of three trait syndromes – armature, mode of fruit dehiscence and aspects of floral heteromorphy – to demonstrate the extent of homoplasy and to show how the repeated evolution of distinctive types of, for example, fruit dehiscence has misled generic delimitation.

Fruits are highly diverse across Caesalpinioideae reflecting adaptations for hydrochory, anemochory, endozoochory, ornithochory, and myrmecochory, as well as several forms of mechanical seed dispersal via explosively, elastically and inertly dehiscent fruits. Here, we show that fruit dehiscence type shows extensive homoplasy across the mimosoid clade, with repeated evolution of, for example, pods elastically dehiscent from the apex, craspedia and lomentiform fruits (Fig. 13). It is now clear that repeated, potentially convergent evolution of fruit types has repeatedly misled generic delimitation and provided the basis for ‘fruit genera’ that have subsequently been shown to be non-monophyletic.

For example, as pointed out by Barneby (1998), the only character uniting Bentham’s (1875) broadly circumscribed pantropical *Calliandra* was the elastically dehiscent fruit, opening from the apex with the valves recurving, but not laterally twisting (Fig. 13a–e). Just how misplaced this reliance on fruit type as a generic synapomorphy was, is evident from the long parade of new genera segregated from *Calliandra*, most of them in the two decades after Barneby (1998) restricted the genus to just the New World species: *Zapoteca* H.M. Hern. (Hernández 1986), *Viguieranthus* Villiers (Du Puy et al. 2002), *Thailentadopsis* Kostermans (Lewis and Schrire 2003), *Afrocalliandra* E.R. Souza & L.P. Queiroz (de Souza et al. 2013) and *Sanjappa* E.R. Souza & M.V. Krishnaraj (de Souza et al. 2016). This procession is still incomplete given that *Calliandra* is still non-monophyletic (Fig. 7), pending phylogenetic placement of the Asian *Calliandraumbrosa* (Wall.) Benth. (see de Souza et al. 2016) and an, as yet, undescribed species (Fig. 7), the last remaining of the species excluded from *Calliandra* by Barneby (1998) that have not yet been placed in a segregate genus. It is clear that the distinctive ‘*Calliandra* pod’ has evolved at least six times independently across Caesalpinioideae (Fig. 13) and occurs in at least 12 phylogenetically scattered genera including *Jaqueshuberia* Ducke, *Bussea* Harms, *Pseudoprosopis* Harms, some species of *Dichrostachys* (DC.) Wight & Arn., *Alantsilodendron* Villiers, *Calliandropsis* H.M. Hern. & P. Guinet, *Calliandra*, *Zapoteca*, *Viguieranthus*, *Sanjappa*, *Afrocalliandra* and a small subset of species of *Acacia*. Of course, it is possible that more detailed anatomical investigation of these morphologically and functionally similar fruits will reveal anatomical differences that show that the homology of this fruit type is misplaced, but the structure of the pod valves and raised sutures of most of these are remarkably similar (Fig. 13a–e).

There are several other examples of classifications and especially genera being misled by parallel evolution of fruit types. For example, the polyphyly of the genus *Enterolobium* Mart. (de Souza et al. 2022a; Figs 10–11) was unexpected because the two clades of *Enterolobium* species share the distinctive indehiscent thickened and curled ‘ear pod’ fruit type. Similarly, it also seems clear that septate lomentiform fruits with valves readily cracking between the seeds and breaking up into one-seeded articles have also evolved multiple times (Fig. 13), often within genera (e.g. Capuron 1970; Aviles et al. 2022; Koenen 2022a; Soares et al. 2022) associated with hydrochory in species adapted to grow in seasonally inundated habitats and this has impacted on generic delimitation. For example, Barneby and Grimes (1996) separated their newly-segregated genera *Balizia* and *Hydrochorea* Barneby & J.W. Grimes on fruit types, yet it is clear that *Hydrochorea* is nested within a paraphyletic *Balizia* (Fig. 9; Soares et al. 2022) and that the distinctive lomentiform fruits of *Hydrochorea* are derived from non-lomentiform indehiscent or follicularly dehiscent pods within this clade (Aviles et al. 2022; Soares et al. 2022). This prevalence of homoplasy associated with fruit types across the mimosoid clade matches that seen across other legume clades (e.g. in subfamily Papilionoideae; Geesink 1984; Hu et al. 2000; Lavin et al. 2001) suggesting that the late developmental stages of the legume pod and associated legume seed dispersal syndromes are prone to convergent evolution, as previously suggested (Geesink 1984; Hu et al. 2000).

Of course, homoplasy per se in no way negates the value and importance of morphology for classification, but instead prompts re-evaluation of homology and the utility of specific morphological characters via reciprocal illumination with new molecular phylogenetic evidence. For example, armature is also homoplasious across Caesalpinioideae with repeated evolution of stipular spines, nodal and internodal prickles, axillary thorns and spinescent shoots (Fig. 15). While armature has been little used as the basis for defining genera because vegetative characters were generally downplayed compared to floral and fruit characters (e.g. Bentham 1875; Burkart 1976), the utility of armature for delimiting some groups within individual clades is increasingly apparent. For example, the four genera segregated from the non-monophyletic *Prosopis* s.l. by Hughes et al. (2022a) are diagnosed by different types of armature (Fig. 15). Similarly, armature is an important character distinguishing the segregates of *Acacia* s.l. (spinescent stipules in *Vachellia*, nodal and internodal prickles in *Senegalia*, unarmed in *Acacia* s.s., *Parasenegalia*, *Pseudosenegalia*, *Mariosousa* and *Acaciella*) and the distribution of prickles (nodal vs. internodal) is discussed in relation to the non-monophyly of *Senegalia* (Terra et al. 2022). Similarly, the two major clades of genera that make up the Caesalpinia Group (Figs 2 and 15) are separated by differences in armature.

Detailed phylogenetic reconstructions for other characters, based on more rigorous and detailed anatomical assessment of homology, will undoubtedly be worthwhile, but it is already clear that the three traits mapped here (Figs 13–15) are not exceptional in terms of their high levels of homoplasy. Leaves also show evolutionarily labile patterns with numerous repeated transitions from micro- to macrophyllidinous leaves within a large majority of Caesalpinioideae genera. Even the more prominent leaf type innovations of bipinnate vs. pinnate leaves, presence of phyllodes and presence or absence of extrafloral leaf nectaries (EFNs) are all hypothesised to be homoplasious. Multiple reversals to once-pinnate leaves within mimosoids (*Inga*, *Calliandrahymenaeodes* (Persoon) Benth., *Sanjappacynometroides* (Bedd.) E.R. Souza & M.V. Krishnaraj and *Cojobarufescens* (Benth.) Britton & Rose), multiple origins of phyllodes (in *Acacia* pro parte, species of *Senna* including *S.phyllodinea* (R. Br.) Symon and some varieties of *S.artemisoides* (Gaudich. ex DC.) Randell and *Mimosa* species including, for example, *M.extranea* Benth. and *M.phyllodinea* Benth. (Barneby 1991)), and multiple losses of EFNs (Marazzi et al. 2019) need to be hypothesised to account for the phylogenetic distributions of these traits. Floral traits show similar extensive homoplasy with multiple derivations of different types of floral heteromorphy (Fig. 14), numerous switches between spikes and capitula and repeated evolution of diverse compound inflorescence conformations (Grimes 1999), homoplasious occurrences of different types of anther glands (Luckow and Grimes 1997) and extremely diverse and evolutionarily labile shapes and sizes of polyads, even within some genera (e.g. Hughes 1997). As indicated above, number of stamens and their connation or not into a staminal tube, the two androecial traits that underpinned the tribal classification of mimosoids first established by Bentham (1875), are also homoplasious across mimosoids such that the tribal classification has not stood the test of time and molecular phylogenetics. Plant functional traits including nodulation (de Faria et al. 2022) and growth forms (Gagnon et al. 2019) also show high levels of homoplasy. Indeed, it appears that nearly all Caesalpinioideae morphological characters and functional traits are homoplasious, given that collectively we, as authors familiar with Caesalpinioideae, have been unable to come up with any morphological characters or functional traits that provide robust synapomorphies subtending larger subclades within Caesalpinioideae, due to either multiple evolutionary origins or repeated independent losses or reversals. Perhaps the one exception to this would be the aquatic habit in *Neptunia* Lour. spp., which is unique within Caesalpinioideae, although many mimosoids are rheophytes, tolerant of seasonal flooding. This is very much in line with the idea that vegetative, flower and fruit characters may be equally homoplasious, as found in other legume groups such as the dalbergioid clade in Papilionoideae (Lavin et al. 2001).

Pre-eminence of certain morphological characters over others in classification of a group and the prevalence of ‘organogenera’ (sensu Nielsen 1981) united by just a single character, in situations where morphology is pervasively homoplasious, has been at the root of many of the disagreements about generic delimitation in mimosoids, as pointed out by Guinet (1981).

### ﻿Trans-continental sampling

A second important reason for the extensive generic non-monophyly is the lack of pantropical synthesis and integration that has been the hallmark of much taxonomic work on Caesalpinioideae up to now and the lack of adequate pantropical sampling of taxa in previous phylogenies. In this light, it is notable that two of the most productive and influential mimosoid taxonomists of the twentieth century, both of whom significantly reshaped the generic classification – Rupert Barneby and Ivan Nielsen – worked largely independently in different geographical areas, especially on genera of the former tribe Ingeae. While both were very much aware of the wider pantropical dimensions and elements of their groups, Barneby focused primarily on New World mimosoids (e.g. Barneby 1991, 1998; Barneby and Grimes 1996, 1997), while Nielsen concentrated on Australasian mimosoids (e.g. Nielsen 1981, 1992) and neither was fully familiar with the details of species of the other (see e.g. Barneby and Grimes 1996), such that no pantropical synthesis across mimosoids was fully achieved and New World – Old World clades that span the Old World and New World or conversely, amphi-Atlantic genera that are non-monophyletic, although hypothesised by both authors, were not resolved.

Our new phylogeny with its near-complete generic sampling reveals several instances of Old World – New World connections and disconnects that have important implications for generic delimitation and which were not fully apparent before. First, the amphi-Atlantic genus *Prosopis* is shown to be non-monophyletic (Figs 4 and 5), confirming earlier evidence of Catalano et al. (2008). *Prosopisafricana* (Guill. & Perr.) Taub. forms a monospecific lineage unrelated to the rest of *Prosopis*, while the remaining three Old World species are sister to the Indo-Nepalese *Indopiptadenia* Brenan and New World *Prosopis* has the Namibian-Namaqualand monospecific *Xerocladia* Harv. nested within it (Fig. 5). It is, therefore, clear that Burkart’s (1976) broad trans-continental concept of *Prosopis* s.l., which followed Bentham’s (1842, 1875) circumscription, is not sustainable (see Hughes et al. 2022a). A second example of disconnection between Old and New World elements of a pantropical genus is *Albizia*, where species of New World section Arthrosamanea (Britton & Rose) Barneby & J.W. Grimes form a clade quite separate from Old World *Albizia* s.s. (Figs 9 and 10; Koenen et al. 2020b: see Aviles et al. 2022). Conversely, two previously poorly understood New World – Old World connections have been revealed. First, it is now clear that the African rainforest species *Albiziaobliquifoliolata* De Willd. and *A.rhombifolia* Benth. (previously often referred to the genus *Cathormion*) are nested within the New World *Balizia* / *Hydrochorea* clade (Fig. 9), which is the focus of generic re-delimitation by Soares et al. (2022). Similarly, the recently segregated Neotropical *Robrichia* (formerly EnterolobiumsectionRobrichia – see de Souza et al. 2022a) is sister to a clade of African mainly rainforest species (*Albiziadinklagei* (Harms) Harms / *A.altissima* Hook. f. / *A.eriorhachis* Harms / *A.leptophylla* Harms) whose generic placements in *Albizia*, *Cathormion* or *Samanea* (Benth.) Merr. have long been uncertain and neglected (Fig. 11), also prompting further generic re-arrangement in this Special Issue by Koenen (2022a). For the first time, the pantropical sampling employed here is more fully documenting these issues.

### ﻿The mimosoid clade

We recover both *Chidlowia* and *Sympetalandra* as firmly nested in the mimosoid clade (Fig. 4), confirming previous molecular phylogenetic studies (*Chidlowia*: Manzanilla and Bruneau 2012; LPWG 2017; Koenen et al. 2020b; *Sympetalandra*: LPWG 2017). Of the ten genera previously included in the Dimorphandra group (sensu Polhill and Vidal 1981), *Sympetalandra*, comprising five species (van Steenis 1975; Hou 1996) in the forests of Malaya, Borneo, the Philippine Islands and the Lesser Sunda Islands, is unique in having its stamens shortly joined to the petals and *Chidlowia* Hoyle (Hoyle 1932) from West Africa (Sierra Leone to Ghana) stands out by having dorsifixed (rather than basifixed) anthers. These two genera are placed between the Xylia and Entada clades of the early-diverging lineages of the mimosoid clade (Fig. 4), outside the core mimosoid clade sensu Koenen et al. (2020b). For *Chidlowia*, once-pinnate leaves and relatively large flowers with showy red petals which are strongly imbricate in bud are more suggestive of placement outside the mimosoids. For example, Hoyle (1932) suggested an affinity with the detarioid genus *Schotia* Jacq., but the regular flowers with equally-sized petals, the showy red stamen filaments partly joined at the base (they were described as free in the genus protologue (Hoyle 1932)) and the small campanulate, gamosepalous calyces, support placement in the mimosoid clade. The placement of *Sympetalandra* in the mimosoid clade, based on molecular analyses, is supported by its racemose or paniculate inflorescences of small, essentially regular, flowers. Finally, the genus *Dinizia*, which on morphological grounds has sometimes been included in mimosoids in the past (Burkart 1943), is here placed in the grade of genera directly subtending the mimosoid clade, confirming the results of previous molecular phylogenetic studies (Luckow et al. 2005; Bouchenak-Khelladi et al. 2010; Marazzi and Sanderson 2010; Manzanilla and Bruneau 2012; Cardoso et al. 2013; Kyalangalilwa et al. 2013; LPWG 2017; Zhang et al. 2020).

The mimosoid clade, i.e. the subfamily formerly known as the mimosoideae, was traditionally diagnosed by petals valvate, as opposed to imbricate, in bud. Valvate petal aestivation is mostly a reflection of whether or not the flowers are actinomorphic vs. zygomorphic, i.e. as the flowers become radially symmetrical the petals become valvate in bud. Across the non-mimosoid grade of Caesalpinioideae subtending the mimosoid clade, taxa with imbricate and valvate aestivation are phylogenetically intermingled. Although the vast majority of mimosoids do, indeed, have valvate petal aestivation, three exceptions: *Chidlowia* (as indicated above), alongside *Mimozyganthus* Burkart and *Parkia* R.Br., both of which are deeply nested within the mimosoid clade, show imbricate petal aestivation, providing further evidence of the homoplasy of this character. Further work to characterise petal aestivation across all relevant genera of Caesalpinioideae is needed, but it is clear that valvate aestivation does not provide a unique diagnostic synapomorphy for the mimosoid clade.

All other aspects of higher-level relationships are discussed in ALS14 Part 2.

### ﻿Taxonomy in the age of phylogenomics

Once purely the domain of morphological analyses (e.g. Barneby and Grimes 1996, 1997; Barneby 1998), decisions on delimiting and naming taxa have increasingly been based on genes rather than morphology (Muñoz-Rodríguez et al. 2019). Employing a large phylogenomic dataset and explicitly considering numbers of genes that support particular generic configurations contribute to naming taxa that are more likely to be robust to future sampling of additional species and genomic regions and, hence, to taxonomic stability (Orthia et al. 2005; Pfeil and Crisp 2005; Humphreys and Linder 2009). However, use of ever larger phylogenomic datasets also raises questions about how to delimit taxa and especially about how conflict amongst gene trees reflecting the widely different evolutionary histories of different parts of the genome (e.g. Salichos and Rokas 2013; Wang et al. 2019; Jiang et al. 2020; Koenen et al. 2020a, b) should inform delimitation of taxa. For example, what fraction of genes supporting a clade should be used as a cut-off for delimiting taxa? To what extent does it matter if there are alternative topologies that are supported by a substantial fraction of genes, even if that number is lower than the number of genes that supports the ‘main’ topology and what are the classificatory implications when only a small fraction of genes is informative for certain relationships (Shen et al. 2017)? Employing large numbers of genes is also enhancing our ability to identify putative hard polytomies on nodes where all, or almost all, genes lack phylogenetic signal (e.g. Koenen et al. 2020b), raising questions about whether it is justified to delimit multiple segregate genera when the relationships amongst them are unresolved and potentially form a polytomy. Large phylogenomic datasets also highlight cases of cytonuclear discordance even more starkly than before, raising questions about what is the best approach when different genomes (i.e. nuclear, plastid and mitochondrial) have different evolutionary histories, as is often the case (e.g. Bruun-Lund et al. 2017; Thielsch et al. 2017; Lee-Yaw et al. 2019; Rose et al. 2021; Debray et al. 2022)? Finally, we might also ask what, fundamentally, is now the role of morphology in delimiting taxa in the phylogenomic era (Muñoz-Rodríguez et al. 2019)?

The phylogeny of Caesalpinioideae presented here (Figs 2–12) poses many of these questions and provides some possible answers. First, the ubiquity of gene tree conflict found here and more generally in phylogenomics (Salichos and Rokas 2013; Wang et al. 2019; Jiang et al. 2020; Koenen et al. 2020b; Yang et al. 2020), suggests that the presence of conflicting topologies for a particular node alone is not sufficient reason to avoid naming the clade subtended by that node. If many conflicting topologies exist, but none of these occurs at a high frequency amongst the gene trees, low support values are indicative of lack of signal rather than true conflict (Koenen et al. 2020b) and do not need to affect classificatory decisions if there is support for the species tree topology amongst a sizable fraction of the gene trees. The nodes subtending *Macrosamanea* Britton & Rose, *Zygia* and *Inga* (Figs 11 and 12) are good examples of an abundance of conflicting topologies none of which is widespread and the monophyly of these genera is, therefore, not in question (except for a few outlier species of *Zygia* – see Appendix 1). However, if low support for a node in the species tree is caused by an alternative topology that is common across gene trees, the situation is more complex and the clade in question should probably not be named pending further study with additional accessions and genomic regions. The crown node of *Archidendron* (Fig. 8) provides an example of a node with a relatively abundant alternative topology, raising doubts about the monophyly of *Archidendron* (see Appendix 1; Brown et al. 2022). Second, in cases of cytonuclear discordance (as we see across several key nodes that affect decisions about generic delimitation), the smaller size of the plastid dataset and the fact that the chloroplast genome can be considered as a single, albeit large, uniparentally-inherited locus, suggest that, in most cases, nuclear phylogenies provide a more accurate approximation of the true species tree (see Terra et al. 2022).

Finally, despite providing the main (usually sole) source of information for classification for centuries, morphology was rapidly eclipsed as a source of data for phylogeny reconstruction with the advent of molecular data (e.g. Scotland et al. 2003). Nevertheless, despite the dominance of phylogenomic data for building accurate and robust trees, morphology continues to play a central role as a complementary source of evidence for delimiting taxa in the light of monophyly inferred from phylogenomic data (Humphreys and Linder 2009; Gagnon et al. 2016). For example, placement of *Zygiasabatieri* and *Z.inundata* not in a clade with the remainder of *Zygia*, but instead as the sister clade of *Inga* in the nuclear ASTRAL phylogeny (Fig. 12) or in a grade subtending *Inga* in the plastome phylogeny (Suppl. material 3; Ferm et al. 2019), presents several options for delimiting genera: transfer these two species to the genus *Inga*, place both species in a new segregate genus or place each species in separate segregate genera. All three options are valid from the perspective of monophyly, but not from a morphological standpoint, because *Z.sabatieri* and *Z.inundata* have dehiscent pods and *Z.sabatieri* has bipinnate leaves, in contrast to the once-pinnate leaves and indehiscent pods that are diagnostic of the genus *Inga*. From a morphological perspective, it will be preferable to assign *Z.inundata* and *Z.sabatieri* to a new segregate genus rather than to transfer them to *Inga*, thereby retaining the morphological integrity and diagnosability of the genus *Inga* (see Appendix 1). This example demonstrates the important role that morphology continues to play in the era of phylogenomics: not to determine relationships and infer monophyly, but to inform and guide decisions about how to partition a phylogeny into monophyletic taxa (see also Terra et al. 2022 for another example).

### ﻿Conclusions and future work

Here, we present a series of phylogenomic analyses including detailed assessment of gene tree conflict and support that suggest that about one quarter of mimosoid genera are non-monophyletic (Figs 2–12). This new backbone phylogeny, building on the 122-taxon version of Koenen et al. (2020b), provides robust foundations for aligning genera with monophyletic groups across a clade where generic delimitation has long been contentious with starkly contrasting generic systems (Lewis et al. 2005; Brown 2008) and for the higher-level classification presented in *Advances in Legume Systematics* 14, Part 2. The limitations of previous work focused either just on the Old World (e.g. Nielsen 1981, 1992) or just on the New World (e.g. Barneby and Grimes 1996, 1997; Barneby 1998) have become more starkly apparent now that pantropical sampling has been achieved, revealing the non-monophyly of well-known pantropical genera, such as *Albizia* (Koenen et al. 2020b; Aviles et al. 2022) and *Prosopis* (Hughes et al. 2022a), as well as previously unrecognised clades with trans-Atlantic distributions (Soares et al. 2022; Koenen 2022a). Our analyses provide a glimpse of the likely extent of morphological homoplasy (Figs 13–15).

However, despite including 420 taxa in the current analyses, it is clear that additional taxon sampling will be needed to fully resolve all the possible non-monophyly issues within Caesalpinioideae. Several priorities for future research are apparent. First, denser taxon sampling across *Senegalia* and allies is needed to address the unusual dilemmas posed by extreme lack of resolution and cytonuclear discordance surrounding delimitation of the genera across the paraphyletic grade comprising *Senegalia*, *Pseudosenegalia*, *Parasenegalia* and *Mariosousa* (Fig. 7) that are explored here by Terra et al. (2022) who provided a list of priority taxa for future sampling with molecular data. Second, the likely non-monophyly of *Archidendron* (see Brown et al. 2022 and Appendix 1) also remains unresolved with a clear need for additional work, especially as many species are known from incomplete material. *Archidendron* and *Senegalia* are now the largest genera in Caesalpinioideae where doubts remain about their monophyly and delimitation. Third, a much more comprehensively sampled study is needed to address the longstanding non-monophyly of *Dimorphandra* Schott (Fig. 3). Fourth, the generic affinities of *Calliandraumbrosa* (Fig. 7; de Souza et al. 2016) and *Calliandra* sp. nov., the last species removed from *Calliandra* by Barneby (1998) yet to be placed in another genus, remain to be assessed. Finally, the taxonomic implications of the non-monophyly of *Zygia* revealed by Ferm et al. (2019) and confirmed here (Figs 11 and 12) have not yet been addressed. Like *Archidendron*, many species of *Zygia* remain poorly understood.

Furthermore, although there is no evidence that any large clades in Caesalpinioideae are subtended by whole genome duplication (WGD) events (Koenen et al. 2020a), it is clear that polyploidisation events have happened many times more recently, scattered across the phylogeny of Caesalpinioideae, for example in *Leucaena* (Govindarajulu et al. 2011; Bailey et al., in prep.), *Vachellia* and *Mimosa* (Dahmer et al. 2011; Simon et al. 2011). Furthermore, high numbers of gene duplications detected on branches subtending, for example, *Sympetalandra*, *Lemurodendron* Villiers & P. Guinet and *Schleinitzia* Warb. point to possible additional WGDs (Ringelberg et al., unpublished data). More work is needed to understand all these possible polyploidisation events, whether they involved auto- or allopolyploidisation and how such events affect assessments of character evolution, homoplasy and generic delimitation.

Finally, our preliminary assessments of homoplasy (Figs 13–15) notwithstanding, there is a clear need for rigorous analysis and comparison of morphological traits across the subfamily, based on more detailed homology assessment of morphological, developmental and genomic data. Morphological diagnosability of taxa is centrally important, especially for the acceptance of novel taxonomy by the end-users of scientific names, a group that is much larger than that of the scientific taxonomic community. We hope that the new phylogeny presented here can provide the evolutionary framework for future morphological studies that assess character evolution and homoplasy in greater detail.

## ﻿Appendix 1

**Generic non-monophyly in Caesalpinioideae – towards a new generic system for the subfamily**

***Caesalpinia***

Divergent circumscriptions of the genus *Caesalpinia* L. were largely resolved by Gagnon et al. (2016) who reduced *Caesalpinia* to ca. nine species and established a new generic system for the Caesalpinia Group as a whole, with 26 genera plus their ‘Ticanto clade’ (*Caesalpiniacrista* L. and allies) as a putative 27^th^ genus. This 27^th^ genus accounts for the non-monophyly of *Caesalpinia* in our analysis (Fig. 2) with *Caesalpiniacrista* representing the Ticanto clade that is re-instated as a genus in this Special Issue by Clark et al. (2022).

***Dimorphandra***

In line with previous studies (Luckow et al. 2005; LPWG 2017), *Dimorphandra* Schott is non-monophyletic in the nuclear phylogeny (Fig. 3), but robustly supported (99% bootstrap support (BS)) as monophyletic in the plastid tree (Suppl. material 3), indicating cytonuclear discordance. This implies either splitting *Dimorphandra* into two genera or sinking *Mora* Schomb. ex Benth., *Stachyothyrsus* Harms and *Burkea* Benth. into *Dimorphandra* (which predates these other three genera). Evidence suggests splitting *Dimorphandra* as the preferred option. First, the three *Dimorphandra* species sampled here represent the three morphologically delimited subgenera (da Silva 1986) with representatives of these subgenera intermingled with other genera rendering *Dimorphandra* polyphyletic in the legume-wide *matK* phylogeny (LPWG 2017) and *Burkea* and *Mora* are not closely related to *Dimorphandra* in the plastid phylogeny (Suppl. material 3; *Stachyothyrsus* is not included in the plastid analysis). Second, while *Mora* has been included in *Dimorphandra* based on morphological similarities (Sandwith 1932; van Steenis 1975), the two genera differ in floral, seed and pod morphology and have generally been treated as distinct (Sandwith 1932; van Steenis 1975; da Silva 1986). African *Stachyothyrsus* and *Burkea* are morphologically (van Steenis 1975) and geographically distinct from South American *Dimorphandra* and *Mora*. All of this suggests that *Dimorphandra* will need to be split into two genera or potentially three, although the robustly supported sister group relationship between *D.davisii* and *D.macrostachya* (internode certainty 0.77, subtended by a long branch) would perhaps favour two genera, rather than three. Additional taxon sampling, to test the monophyly of the three subgenera, is required before taxonomic re-arrangements can be made. If the genus is to be split, the name Dimorphandrawould remain attached tosubgenusDimorphandra, here represented by *D.gardneriana* Tul. *Dimorphandraexaltata* Schott is the type species of the genus. The names of the other two subgenera, *Phaneropsia* Tulasne and *Pocillum* Tulasne, would be available for the remaining species. Both names originate from the same publication (Tulasne 1844), but since *Pocillum* also refers to a genus of fungi (Kirk et al. 2008), *Phaneropsia* would be the more suitable generic name for the species not in *Dimorphandra* s.s. However, as taxon names have no priority at different rank (Turland et al. 2018), a new generic name may also be proposed.

***Xylia* and *Calpocalyx***

The non-monophyly of *Xylia* with *Calpocalyx* nested within it was documented using *matK* sequences (LPWG 2017) and is confirmed here (Fig. 4). This does not come as a great surprise, as these genera have always been considered closely related (Villiers 1984; Lewis et al. 2005). They have overlapping geographical and ecological distributions mainly in the tropical rainforests of central and western Africa (although *Xylia* has a wider distribution in Africa, Madagascar and Asia). The two genera also share a suite of morphological characteristics (Villiers 1984; Luckow et al. 2003), including robust woody sickle-shaped explosively dehiscent fruits (Fig. 13), a chromosome count of 2n = 12 (Goldblatt and Davidse 1977) and pollen grains in small-sized polyads (Jumah 1991). Since the name *Xylia* (Bentham 1841) predates *Calpocalyx* (Engler and Prantl 1897) and given the morphological and ecological similarities of the two genera, the most straightforward solution to the non-monophyly presented here would be the transfer of the species of *Calpocalyx* to *Xylia*. However, this apparently straightforward incorporation of *Calpocalyx* into *Xylia* is complicated by the name *Esclerona* Raf., an apparently valid name predating *Xylia*, raising the possibility of proposing conservation of the name *Xylia* prior to merging these two genera.

***Entada* and *Elephantorrhiza***

A close relationship between *Entada* Adans. and *Elephantorrhiza* Benth. has long been suggested in all molecular phylogenies that sampled these genera (e.g. Luckow et al. 2003; Koenen et al. 2020b). With denser sampling of species, it has become clear that *Elephantorrhiza* is nested within *Entada* (LPWG 2017), a result that is confirmed here (Fig. 4) and which provides the basis for re-circumscription of *Entada* to include *Elephantorrhiza* by O’Donnell et al. (2022) in this Special Issue.

***Prosopis***

One of the most striking and robustly supported examples of generic non-monophyly in our analyses is *Prosopis* s.l. whose species are placed in four separate lineages (Figs 4 and 5). The nodes supporting this non-monophyly are some of the most robustly supported across the Caesalpinioideae phylogeny as a whole (Fig. 5). This shows that *P.africana* is not closely related to the rest of *Prosopis* s.l., but is placed in a grade with other monospecific or species-poor genera subtending the core mimosoid clade (Fig. 4), confirming results from earlier studies (Catalano et al. 2008; LPWG 2017; Koenen et al. 2020b). The rest of Old World *Prosopis* (three species) is sister to the Indo-Nepalese genus *Indopiptadenia* and New World *Prosopis* has the Namibian – S. African *Xerocladia* nested within it (Fig. 5). A new generic classification of *Prosopis* s.l., accounting for this non-monophyly, is presented in this Special Issue by Hughes et al. (2022a).

***Desmanthus***

The non-monophyly of *Desmanthus* with the monospecific Hawaiian endemic *Kanaloa* Lorence & K.R. Wood nested within it (Fig. 5) mirrors earlier phylogenies (Hughes et al. 2003; Luckow et al. 2003, 2005) and is in line with the morphological distinctiveness of *Desmanthusbalsensis* J.L. Contreras from the remaining species of *Desmanthus* (Contreras Jiménez 1986; Luckow 1993). A new monospecific segregate genus to account for this non-monophyly is proposed in this Special Issue by Hughes et al. (2022b).

***Dichrostachys* , *Gagnebina* and *Alantsilodendron***

*Dichrostachys* (DC.) Wight & Arn. and *Alantsilodendron* Villiers are both recovered as non-monophyletic in our sparsely sampled analysis (Fig. 5), raising questions about the monophyly of *Gagnebina* Neck. ex DC., here represented by just a single species. The Malagasy members of these three genera (all species in our phylogeny, except *D.cinerea* R. Vig.) cluster together in a clade characterised by very short branches and extensive gene tree conflict (Fig. 5) suggestive of an early burst model of diversification typical of a rapid radiation on Madagascar (Aebli 2015). Previous molecular phylogenetic studies have also found at least some of these genera to be non-monophyletic (Hughes et al. 2003; Luckow et al. 2003, 2005; Aebli 2015) and some species have been transferred between genera based on morphology (Lewis and Guinet 1986). Each of these genera contains several other species from Madagascar not sampled here. While a parsimonious solution could be to merge the three genera into *Gagnebina* (de Candolle 1825) (a name predating *Dichrostachys* (Wight and Walker-Arnott 1834) and *Alantsilodendron* (Villiers 1994)), such a move would result in a highly variable genus, with no consistent morphological character to distinguish it. A forthcoming monograph (Luckow, unpublished data) will resolve the non-monophyly of these genera by transferring two species of *Dichrostachys* to *Alantsilodendron* and seven to a new genus (Phillipson et al. 2022). Additional sampling of non-Malagasy species of *Dichrostachys* would also be important, especially Australian *D.spicata*, as it has been placed as sister to the combined *Dichrostachys* / *Gagnebina* / *Alantsilodendron* + *Calliandropsisnervosa* (Britton & Rose) H.M. Hern. & Guinet clade in several studies (Hughes et al. 2003; Luckow et al. 2003, 2005; Aebli 2015). The African species *D.dehiscens* Balf. f. and *D.kirkii* Benth. also need to be sampled as they share a dehiscent fruit type with members of the new Madagascan genus.

***Stryphnodendron* and *Pseudopiptadenia***

Our analyses support the monophyly of the Stryphnodendron clade sensu Koenen et al. (2020b) comprising the genera *Parapiptadenia* Brenan, *Pityrocarpa* (Benth. & Hook.f.) Britton & Rose, *Pseudopiptadenia* Rauschert and *Stryphnodendron* Mart. (Fig. 6) and presumably *Microlobius* C. Presl., which, although not sampled here, has been shown to be nested within or sister to *Stryphnodendron* (Ribeiro et al. 2018; Simon et al. 2016; see also Lima et al. 2022). Of these genera, only *Parapiptadenia* is monophyletic in our analyses, although *Pityrocarpa* is here only represented by a single taxon (Fig. 6). *Stryphnodendron* is non-monophyletic as *S.duckeanum* Occhioni does not group with the rest of the genus (Fig. 6), in line with flower, fruit and branching characteristics that suggested transfer of *S.duckeanum* to another genus (Scalon 2007) and with previous molecular phylogenies showing *S.duckeanum* separated from the rest of *Stryphnodendron* (Jobson and Luckow 2007; Simon et al. 2016; Ribeiro et al. 2018; Sauter 2019). Similarly, *Pseudopiptadenia* is also non-monophyletic with *P.schumanniana* placed as sister to the single sampled species of *Pityrocarpa*, rather than forming a clade with *Pseudopiptadeniacontorta* (DC.) G.P. Lewis & M.P. Lima and *P.psilostachya* (DC.) G.P. Lewis & M.P. Lima (Fig. 6). Several previous molecular phylogenies also found *Pseudopiptadenia* to be non-monophyletic – however, those studies did not include *P.schumanniana* and found *P.brenanii* G.P. Lewis & M.P. Lima (not sampled here) to be the outlier instead (Simon et al. 2016; Ribeiro et al. 2018). The sparsely sampled backbone phylogeny of the Stryphnodendron clade presented here provides the foundations for more densely sampled analyses and re-delimitation of both *Stryphnodendron* (Lima et al. 2022) and *Pseudopiptadenia* / *Pityrocarpa* (Borges et al. 2022) in this Special Issue.

The remaining genera in the Stryphnodendron and Mimosa clades are all monophyletic (Fig. 6), confirming previous phylogenetic studies and taxonomic rearrangements, including segregation of *Lachesiodendron* P.G. Ribeiro, L.P. Queiroz & Luckow from *Piptadenia* (Ribeiro et al. 2018), as well as placement of amphi-Atlantic *Adenopodia* C. Presl as sister to *Mimosa* and the sister group relationships amongst the main clades of *Mimosa* (Simon et al. 2011).

***Senegalia* and allied genera**

The striking cytonuclear discordance whereby *Senegalia* Raf. appears as non-monophyletic in the analyses of nuclear gene sequences, but as monophyletic in the analyses of plastomes, was first revealed by Koenen et al. (2020b), a result confirmed here by sampling more species of *Senegalia*, plus the closely related *Mariosousa*, *Parasenegalia* and *Pseudosenegalia* (Fig. 7). In the nuclear gene analyses, the two clades of *Senegalia* plus these three other genera and the incompletely known *Albizialeonardii* Britton & Rose ex Barneby & J.W. Grimes form a paraphyletic grade with very short and poorly supported or unsupported internal branches (Fig. 7). The complex and intriguing issues these features raise for delimitation of *Senegalia* are explored by Terra et al. (2022), who conclude that sequencing of more species is required.

***Calliandra***

Following reduction of Bentham’s (1875) broad trans-continental circumscription of *Calliandra* Benth. to just the New World species by Barneby (1998), five genera have been segregated to account for the majority of the Old World species. Now just a handful of Old World species remain to be resolved, including the Asian *Calliandra* sp. nov. (*Poilane 9150*), that, as expected, does not group together with the New World *Calliandra* s.s., but is instead sister to the Indian monospecific genus *Sanjappa* É.R. Souza & M.V. Krishnaraj in the Zapoteca clade (Fig. 7). Bentham (1875) included four Asian species in *Calliandra* (de Souza et al. 2013), which share the apically dehiscent pods of *Calliandra* (Fig. 13a–f), but in other respects present anomalies, especially in the configuration of their polyads. The identities of these Asian *Calliandra* species have long been considered ambiguous (Barneby 1998). Two of these Asian species have been assigned to different genera (*C.cynometroides* Bedd. to *Sanjappa* (de Souza et al. 2016) and *C.geminata* (Wight & Arn.) Benth. to *Thailentadopsis* Kosterm. (Lewis and Schrire 2003)), while the generic placement of the remaining species, *C.umbrosa* (Wall.) Benth., remains unknown. The fourth species, *C.griffithii* Baker ex Benth., is now considered a subspecies of *C.umbrosa* (Paul 1979). *Calliandraumbrosa* has never been included in a molecular phylogenetic analysis (de Souza et al. 2013, 2016) and, unfortunately, sequencing of *C.umbrosa* was unsuccessful in this study. However, polyad, leaf, corolla and pod morphology, plus the presence of facultatively spinescent stipules, distinguish *C.umbrosa* from other genera, suggesting that it should potentially be assigned to a new genus (de Souza et al. 2013, 2016). Until DNA sequences of *C.umbrosa* can be obtained to ascertain its relationship to *Calliandra* sp. nov., this residual non-monophyly of the genus *Calliandra* cannot be resolved.

***Pithecellobium* and allies**

While the Pithecellobium alliance is the only one of the informal alliances of Barneby and Grimes (1996) whose monophyly has withstood the test of phylogenomic analysis (Koenen et al. 2020b), other than *Pithecellobium* Mart. itself, our sparsely sampled phylogeny of this clade suggests that the monophyly of the four other genera placed in the Pithecellobium clade (*Painteria* Britton & Rose, *Havardia* Small, *Ebenopsis* Britton & Rose and *Sphinga* Barneby & J.W. Grimes) is doubtful and needs to be further tested with more complete taxon sampling (Fig. 7). Even with our limited taxon sampling, *Painteria* and *Havardia* are clearly non-monophyletic (Fig. 7), raising significant doubts about the taxonomic status of *Ebenopsis* and *Sphinga*, which are both represented by only one species in our trees. *Painteria* is especially poorly distinguished from *Havardia*; *Sphinga* was originally described in *Havardia* and previous studies (Nielsen 1981; Polhill 1994) placed all four genera in a more broadly defined *Havardia* (Brown 2008). Such a solution might, therefore, seem sensible, but together they form a paraphyletic grade in our phylogenies (Fig. 7), suggesting that unless all four genera were to be sunk back into *Pithecellobium* (from which they were segregated (Barneby and Grimes 1996)), these four genera require at least three names, as they are divided over three (poorly-supported) lineages: one comprising *Spingaacatlensis* (Benth.) Barneby & J.W. Grimes and *Havardiacampylacantha* (L. Rico & M. Sousa) Barneby & J.W. Grimes, one *Painterialeptophylla* (DC.) Britton & Rose, *Pa.elachistophylla* (A. Gray ex S. Watson) Britton & Rose and *Ebenopsisconfinis* (Standl.) Britton & Rose and one *H.pallens* (Benth.) Britton & Rose, which is the type species of *Havardia* and sister to *Pithecellobium*. Clearly, taxon sampling in our phylogeny is too limited to draw firm taxonomic conclusions. A new phylogeny of the Pithecellobium clade, presented here in this Special Issue, is used as the basis for erecting two new genera to account for these generic non-monophyly issues (Tamayo-Cen et al. 2022). This new phylogeny, based on a small set of DNA sequence loci, but with denser taxon sampling than that encompassed here, is not fully congruent with the phylogenomic backbone presented in Fig. 7.

**The Archidendron clade**

The genera and lineages of the large Archidendron clade comprising *Acacia* Mill., *Archidendron* F. Muell. and six smaller genera (Fig. 8; Koenen et al. 2020b), together make up over one third of all mimosoid species and are restricted to Australasia. Relationships across the backbone of this clade are complex and generally poorly resolved with very short branches and high levels of gene tree conflict and lack of phylogenetic signal across a significant fraction of genes (Fig. 8), such that the topologies across different analytical approaches can differ. This suggests that some nodes across this backbone should better be viewed as putative polytomies. Three genera in this clade, *Wallaceodendron* Koord., *Pararchidendron* I.C. Nielsen and *Paraserianthes* I.C. Nielsen, are monospecific. *Falcataria* (I.C. Nielsen) Barneby & J.W. Grimes comprises three species but is represented by only one taxon in our phylogeny, so no conclusion can, therefore, be made about its monophyly, although our results support the segregation of this genus from *Paraserianthes* (Barneby and Grimes 1996; Brown et al. 2011). Three of the four remaining genera are monophyletic: *Acacia*, *Archidendron* and *Serianthes* Benth. (confirming the results of Demeulenaere et al. (2022) in this Special Issue). However, the monophyly of *Archidendron* remains doubtful as it is supported by few gene trees and opposed by many (Fig. 8) and the genus is not monophyletic in the plastid tree (Suppl. material 3). This is very much in line with previous findings of a non-monophyletic *Archidendron* (Brown et al. 2008, 2011; Iganci et al. 2016; LPWG 2017). The likely non-monophyly of *Archidendron* is explored in more detail in this Special Issue by Brown et al. (2022). It is notable that the two well-supported *Archidendron* subclades found here are replicated by Brown et al. (2022), where their morphological and geographical identities are discussed in detail. Finally, the non-monophyly of *Archidendropsis* I.C. Nielsen, documented and addressed in this Special Issue by Brown et al. (2022), is confirmed by the much larger phylogenomic dataset analysed here (Fig. 8).

Our results weakly support *Paraseriantheslophantha* as sister to *Acacia* (Fig. 8), in line with earlier findings (Brown et al. 2008, 2011; Koenen et al. 2020b) and shared morphological similarities including hard seeds that are stimulated to germinate by fire (Brown et al. 2011), minute anthers and numerous stamens (Barneby and Grimes 1996). As *P.lophantha* contains two geographically disjunct subspecies, P.lophanthasubsp.montana (Jungh.) I.C. Nielsen in Indonesia and P.lophanthasubsp.lophantha (the subspecies sequenced here) in southern Australia (Brown et al. 2011), sequencing the missing subspecies would be worthwhile to check that the two cluster together as sister to *Acacia*. However, it is important to note that this relationship is sensitive to the type of dataset and phylogenetic method: the ASTRAL trees (Fig. 8) recover *P.lophantha* as the sister of *Acacia*, whereas the nuclear RAxML phylogenies (Ringelberg et al. 2022) find a sister relationship between *Acacia* and *Archidendron* plus *Archidendropsisxanthoxylon* (C.T. White & W.D. Francis) I.C. Nielsen, the PhyloBayes gene jack-knifing phylogeny (Ringelberg et al. 2022) resolves the whole Archidendron clade as one large polytomy lacking a clear sister lineage to *Acacia* and the plastid tree (Suppl. material 3) recovers *Archidendropsisxanthoxylon* as sole sister of *Acacia*. Furthermore, *P.lophantha* and several species of *Archidendron* are also identified as species often changing positions across trees by RogueNarok (Aberer et al. 2013). The high levels of intergenic conflict, very short branches, extremely low bootstrap support values especially in the nucleotide RAxML phylogenies, lack of concordance and signal amongst the gene trees and failure to reject a polytomy by ASTRAL (Fig. 8), all suggest that the backbone of the Archidendron clade should perhaps best be viewed as one large polytomy, as depicted in the PhyloBayes consensus tree (Ringelberg et al. 2022). However, the number (eight in the PhyloBayes phylogeny) and precise identity of lineages arising from this tangle remain unclear and relationships amongst the genera of this clade remain highly uncertain pending additional taxon sampling and detailed investigation of the causes of gene tree conflict and possible evidence for introgression.

***Albizia***

At the start of this study, the genus *Albizia* was dubbed the last pantropical so-called ‘dustbin’ genus pending resolution (Koenen et al. 2020b). Here, we show that *Albizia* s.l. is rampantly non-monophyletic, most notably because the bulk of the Old and New World species are placed in separate clades (Figs 9 and 10). This Old World – New World split is remedied in this Special Issue by Aviles et al. (2022) who resurrect the genus *Pseudalbizzia* Britton & Rose for the majority of the New World species placed in Barneby’s AlbiziasectionArthrosamanea, with *Albizia* s.s. now restricted to just the Old World species, which still includes ca. 90 spp. (Koenen et al., unpubl. data). Furthermore, the disparate placements of several other species of *Albizia* across the phylogeny, viz: *Albiziacarbonaria* Britton (Fig. 8), the long-neglected African *Albizia* species previously often placed in *Cathormion* or *Samanea* (Benth.) Merr. (Figs 9 and 11) and *Albizialeonardii* (Fig. 7), are all accounted for with new generic placements and nomenclatural combinations (Koenen 2022b; Soares et al. 2022), one synonymisation (Terra et al. 2022) and a new segregate genus (Koenen 2022a), all of them being published in this Special Issue.

***Abarema* , *Hydrochorea* and *Balizia***

The recent re-circumscription of *Abarema* Pittier to include just two species and transfer of the remaining species to the re-instated *Punjuba* Britton & Rose and *Jupunba* Britton & Rose (Guerra et al. 2016, 2019; Iganci et al. 2016; Soares et al. 2021), is broadly supported here (Figs 9 and 11), except for the anomalous placement of *Jupunbamacradenia* (Pittier) M.V.B. Soares, M.P. Morim & Iganci which is sister to the *Hydrochorea* + *Balizia* clade (Fig. 9). This placement is unexpected and somewhat suspect considering *J.macradenia* is firmly placed in *Jupunba* in Soares et al. (2021). As found by Iganci et al. (2016), Koenen et al. (2020b) and Soares et al. (2021), *Balizia* is non-monophyletic with the genus *Hydrochorea* plus two African species of *Albizia* nested within it (Fig. 9). *Hydrochorea* is re-circumscribed to accommodate all these elements by Soares et al. (2022) in this Special Issue.

***Leucochloron***

Koenen et al. (2020b) showed that *Leucochloron* is polyphyletic and that result is confirmed here, split between the Albizia and Inga clades (Figs 10 and 11). A new segregate genus to account for this non-monophyly is proposed in this Special Issue by de Souza et al. (2022b).

***Zygia* , *Macrosamanea* and *Inga***

Alongside *Archidendron*, the large Neotropical, mainly rainforest genus *Zygia* remains one of the least well-documented genera of mimosoids, with many species known from incomplete material (Barneby and Grimes 1997). Previous work by Ferm et al. (2019) showed that, while the bulk of genus *Zygia* is monophyletic, a handful of outlier species have affinities to other genera: *Zygiaocumarensis* (Pittier) Barneby & J.W. Grimes is sister to *Macrosamanea* Britton & Rose ex Britton & Killip, *Marmaroxylonmagdalenae* Killip ex. L. Rico (treated as a synonym of *Z.ocumarensis* by Barneby and Grimes (1997)) is nested in *Jupunba* and *Z.inundata* and *Z.sabatieri* are together sister to *Inga*. With the exception of *M.magdalenae*, which is not included in this study, these placements are confirmed here with phylogenomic data (Figs 11 and 12) and reflect the morphological distinctiveness of these species from the rest of the genus (Barneby and Grimes 1997; Ferm et al. 2019) which prompted placements in their own separate monospecific sections of *Zygia* (Barneby and Grimes 1997). New nomenclatural combinations to deal with these outlier *Zygia* species are still pending. We suggest that *Zygiaocumarensis* should best be transferred to *Macrosamanea*, as it shares bipinnate leaves with multiple pairs of pinnae and an absence of cauli-/ramiflory (which is almost universal in *Zygia*) with several species of *Macrosamanea* (Barneby and Grimes 1996; Ferm et al. 2019). The identity of *Marmaroxylonmagdalenae* needs to be re-evaluated, but the evidence of Ferm et al. (2019), who sampled the type material, suggests it should be transferred to *Jupunba*. The generic placements of *Z.inundata* and *Z.sabatieri* are more contentious. Arguments can be made to transfer *Z.inundata* to *Inga* (Ferm et al. 2019): it was originally described in *Inga* and it shares once-pinnate leaves and absence of cauli-/ramiflory with *Inga* (Barneby and Grimes 1997; Ferm et al. 2019). However, *Z.inundata* was placed as the sole sister of *Inga* in the plastid tree (Suppl. material 3) and by Ferm et al. (2019), whereas the nuclear gene data suggest that *Z.inundata* is sister to *Z.sabatieri* and together these two species form the sister clade of *Inga* (Fig. 12). *Zygiasabatieri* has bipinnate leaves and both *Z.sabatieri* and *Z.inundata* have dehiscent pods, characteristics that distinguish these species from *Inga* with its uniformly once-pinnate leaves and indehiscent pods. In order to maintain a morphologically coherent and homogeneous *Inga* with respect to these diagnostic characters, segregating *Z.inundata* and *Z.sabatieri* as a new genus would appear to be advantageous. *Ingopsis* Barneby & J.W. Grimes and *Pseudocojoba* Barneby & J.W. Grimes, the names for the monospecific sections containing *Z.inundata* and *Z.sabatieri*, respectively (Barneby and Grimes 1997), are two available names, of which *Ingopsis* would be preferable given the morphological and phylogenetic proximity of this clade to *Inga* and the lack of a close relationship to *Cojoba* Britton & Rose. However, since these sectional names have no priority at generic rank (Turland et al. 2018), alternatively, a new name could equally be proposed. Finally, while *Zygia* s.s. was reasonably well sampled by Ferm et al. (2019) and also in the current study (Fig. 12), alongside further herbarium taxonomic work and field studies to clarify species, denser phylogenetic taxon sampling is desirable, in particular to include *Z.eperuetorum* (Sandwith) Barneby & J.W. Grimes. This species is known only from the Essequibo Valley in Guyana, was placed in its own section by Barneby and Grimes (1997), has an unusual combination of morphological characters not found elsewhere in *Zygia* and the fruit remains unknown. *Zygiaeperuetorum* may well, therefore, represent an additional separate lineage that could potentially merit recognition as a distinct genus.

## Supplementary materials

Supplementary material 1 Table S1 Data type excel file. Explanation note Samples included in this study. https://binary.pensoft.net/file/732560This dataset is made available under the Open Database License (http://opendatacommons.org/licenses/odbl/1.0/). The Open Database License (ODbL) is a license agreement intended to allow users to freely share, modify, and use this Dataset while maintaining this same freedom for others, provided that the original source and author(s) are credited.Jens J. Ringelberg, Erik J.M. Koenen, João R. Iganci, Luciano P. de Queiroz, Daniel J. Murphy, Myriam Gaudeul, Anne Bruneau, Melissa Luckow, Gwilym P. Lewis, Colin E. Hughes

Supplementary material 2 Table S2 Data type excel file. Explanation note Trait data used for character evolution analyses. https://binary.pensoft.net/file/732561This dataset is made available under the Open Database License (http://opendatacommons.org/licenses/odbl/1.0/). The Open Database License (ODbL) is a license agreement intended to allow users to freely share, modify, and use this Dataset while maintaining this same freedom for others, provided that the original source and author(s) are credited.Jens J. Ringelberg, Erik J.M. Koenen, João R. Iganci, Luciano P. de Queiroz, Daniel J. Murphy, Myriam Gaudeul, Anne Bruneau, Melissa Luckow, Gwilym P. Lewis, Colin E. Hughes

Supplementary material 3 Figure S1 Data type Pdf file. Explanation note Chloroplast phylogeny of Caesalpinioideae. Only bootstrap support values lower than 100% are shown. https://binary.pensoft.net/file/732562This dataset is made available under the Open Database License (http://opendatacommons.org/licenses/odbl/1.0/). The Open Database License (ODbL) is a license agreement intended to allow users to freely share, modify, and use this Dataset while maintaining this same freedom for others, provided that the original source and author(s) are credited.Jens J. Ringelberg, Erik J.M. Koenen, João R. Iganci, Luciano P. de Queiroz, Daniel J. Murphy, Myriam Gaudeul, Anne Bruneau, Melissa Luckow, Gwilym P. Lewis, Colin E. Hughes

Supplementary material 4 Supplementary tree file Data type Tree file (Newick format). Explanation note Tree file of the ASTRAL phylogeny based on the single-copy genes (depicted in Figs 2–12), in which taxon labels have been updated to reflect taxonomic changes made in all the entries in Advances in Legume Systematics 14 Part 1. https://binary.pensoft.net/file/732563This dataset is made available under the Open Database License (http://opendatacommons.org/licenses/odbl/1.0/). The Open Database License (ODbL) is a license agreement intended to allow users to freely share, modify, and use this Dataset while maintaining this same freedom for others, provided that the original source and author(s) are credited.Jens J. Ringelberg, Erik J.M. Koenen, João R. Iganci, Luciano P. de Queiroz, Daniel J. Murphy, Myriam Gaudeul, Anne Bruneau, Melissa Luckow, Gwilym P. Lewis, Colin E. Hughes
