# Supplementary figures and images for: ﻿Phylogenomic analysis of 997 nuclear genes reveals the need for extensive generic re-delimitation in Caesalpinioideae (Leguminosae)
Source: PhytoKeys. 2022 Aug 22;205:3–58. doi: 10.3897/phytokeys.205.85866 (PMC9848904; doi:10.3897/phytokeys.205.85866)

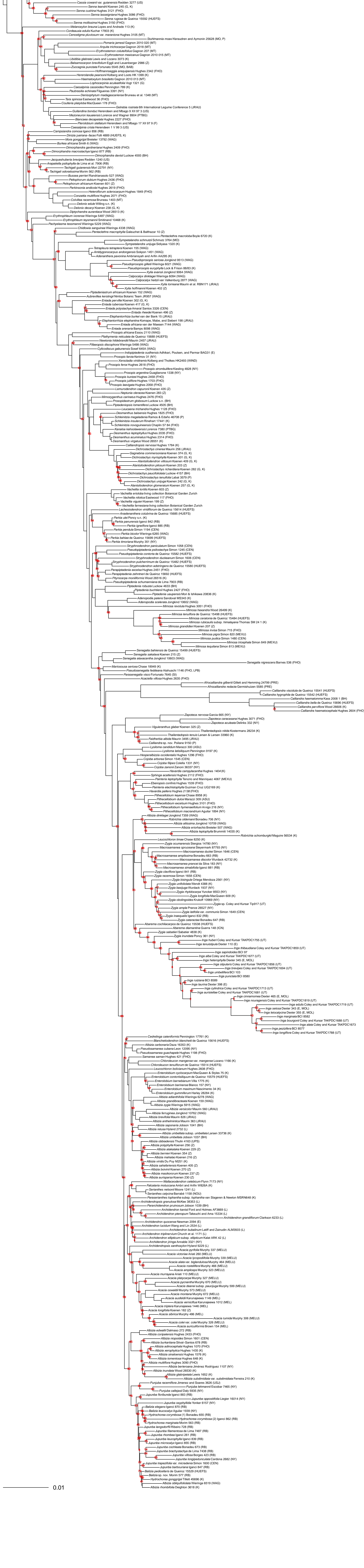

Supplement: Supplementary material 3 — Figure S1 [file phytokeys-205-003_article-85866__-s003.pdf]
